# Artificial intelligence to investigate metabolomics data for precision medicine

**DOI:** 10.1007/s11306-026-02401-z

**Published:** 2026-02-27

**Authors:** Antony Shenouda, Sahana Senthilkumar, Youssef Mourad, Joy Xie, Elizabeth Peker, Saman Zeeshan, Zeeshan Ahmed

**Affiliations:** 1 Rutgers Institute for Health, Health Care Policy and Aging Research, Rutgers, The State University of New Jersey, 112 Paterson Street, New Brunswick, NJ 08901 USA; 2 Department of Biomedical and Health Informatics, UMKC School of Medicine, 2411 Holmes Street, Kansas City, MO 64108 USA; 3 Department of Medicine, Division of Cardiovascular Diseases and Hypertension, Robert Wood Johnson Medical School, Rutgers Health, 125 Paterson St, New Brunswick, NJ 08901 USA

**Keywords:** Artificial Intelligence, Machine Learning, Metabolomics, Therapeutic, Precision Medicine

## Abstract

**Background:**

Metabolomic data offers insights into disease mechanisms, diagnostics, and therapeutic targets by analyzing metabolic profiles. In analyzing these profiles, traditional bioinformatic and statistical approaches, while valuable, often struggle to process high-dimensional and nonlinear metabolic data, lacking the sensitivity and adaptability that artificial intelligence (AI) and machine learning (ML) techniques provide. The integration of AI/ML has greatly enhanced the metabolomics field, enabling biomarker identification, disease prediction, and classification of metabolic patterns at an unprecedented level.

**Aim of review:**

This study analyses and compares the scientific goals, methodologies, datasets, and sources of AI/ML approaches applied to metabolomic data, as well as assessing their implications in precision medicine. We systematically reviewed recent advancements in AI/ML applications to metabolomic data, focusing on peer-reviewed research indexed in PubMed. Significant number of studies were analyzed, covering diseases such as cancer, cardiovascular diseases, and diabetes. Our results showed that the most used AI/ML techniques were SVM, RF, Gradient Boosting, and Logistic Regression, highlighting their effectiveness in processing complex metabolic data. Despite these advancements, key challenges persist in AI/ML applications to metabolomics data, including small cohort sizes, data heterogeneity, and the need for improved model interpretability, and these challenges must be considered for future use.

**Key scientific concepts of review:**

Ultimately, our findings underscore the transformative potential of AI/ML in metabolomics and its critical role in advancing precision medicine by uncovering novel metabolic pathways, improving treatment strategies, and enabling the earlier diagnosis of diseases through predictive metabolic profiling.

## Introduction

Metabolomics has emerged as a powerful tool in understanding disease mechanisms, improving diagnostics, and identifying potential therapeutic targets. It plays a vital role in assessing disease states, diagnosing conditions, and uncovering underlying biochemical processes through the study of metabolites in the human body (Clish, 2015). By analyzing the levels of these metabolites, a detailed understanding of metabolic activity in both healthy and diseased states can be revealed. Differences in metabolite profiles not only help identify and differentiate between these states but also enable the detection of sensitive biomarkers (Clish, 2015). These biomarkers can guide more accurate diagnoses and predict disease risk (Roberts & Gerszten, 2013). This ability makes metabolomics a critical component of precision medicine, as it helps tailor treatments to the unique metabolomic makeup of each patient, ultimately improving treatment outcomes and enhancing patient care (Clish, 2015). Thus, it is not surprising that metabolomics has been studied in a multitude of diseases, including but not limited to cardiovascular diseases, cancers, and diabetes.

Metabolites, which include a wide range of small molecules such as proteins, carbohydrates, lipids, amino acids, and nucleotides, can be classified as endogenous (originating from within the body), or exogenous (derived from external sources such as diet, drugs, and environmental exposures) (Kong & Hernandez-Ferrer, 2020). Metabolic data can be acquired using either targeted or untargeted approaches, where targeted metabolomics (Roberts et al., 2012) focuses on quantifying specific, predefined metabolites, and untargeted metabolomics (Vinayavekhin & Saghatelian, 2010) aims to broadly profile all detectable metabolites in a sample without prior bias. Several techniques are employed for these analyses, with the most common being Liquid Chromatography-Tandem Mass Spectrometry (LC–MS) (Seger & Salzmann, 2020). This method combines liquid chromatography, which separates compounds based on their chemical properties, with mass spectrometry, which identifies and quantifies the compounds by measuring their mass-to-charge (m/z) ratio (Thomas et al., 2022). A variant of LC–MS, Ultra-Performance Liquid Chromatography-Mass Spectrometry (UPLC-MS), utilizes smaller particles in the chromatographic column to achieve higher resolution, faster analysis, and greater sensitivity (Fernández & Samyn, 2011). Another widely used method is Gas Chromatography-Tandem Mass Spectrometry (GC–MS), which separates volatile compounds (compounds with high vapor pressure) using gas chromatography, followed by mass spectrometry for detection and analysis of metabolites (Garcia & Barbas, 2011). Capillary Electrophoresis-Mass Spectrometry (CE-MS) is another technique, which separates charged metabolites based on their electrophoretic mobility (velocity of charged particles in an electric field) and subsequently analyzes them using mass spectrometry (Zhang & Ramautar, 2021). NMR-Derived Metabolic Profiles utilize Nuclear Magnetic Resonance (NMR) Spectroscopy to detect the magnetic properties of atomic nuclei in a strong magnetic field, providing structural and quantitative information about metabolites (Cerulli et al., 2021). Lastly, Single-Cell Raman Spectroscopy measures the scattering of light by molecules, offering a unique molecular fingerprint (Li et al., 2012). This technique enables metabolite identification at the single-cell level by analyzing their vibrational modes.The variety of differing data acquisition techniques allows for metabolites to be captured from various sources and sample amounts, enabling further downstream analysis.

Analyzing metabolic data involves a range of approaches, including bioinformatic, statistical, and, more recently, Artificial Intelligence (AI) and Machine Learning (ML) methods. Bioinformatic approaches involve the exploration and interpretation of biological data using specialized algorithms and computational techniques (Chen et al., 2015). These approaches encompass various stages, such as data preprocessing, integration across different sets of biological molecules (omics layers), and pathway analysis. An example of pathway analysis is the use of the Kyoto Encyclopedia of Genes and Genomes (KEGG) database, which maps biological data onto established pathways to provide insights into metabolic processes, while also highlighting the intricate interactions between various omics such as genomics, proteomics, and metabolomics, to uncover deeper biological insights (Chen et al., 2015). Statistical methods, such as Principal Component Analysis (PCA) (Groth et al., 2013) and Analysis of Variance (ANOVA) (Kim, 2017) are widely employed to identify trends and draw conclusions from metabolic data. However, both bioinformatics and statistical techniques often struggle to address the complexity and scale of large metabolic datasets (Jamialahmadi et al., 2024). In recent years, AI/ML approaches have emerged as transformative tools in metabolomic research due to their advanced predictive and analytical capabilities, far beyond the reach of traditional statistical and bioinformatic methods (Jamialahmadi et al., 2024). These technologies excel in uncovering intricate patterns and explaining nonlinear relationships within vast datasets. AI/ML has enabled the development of highly effective models for applications like disease classification and biomarker discovery, significantly advancing the metabolomics field and knowledge about disease etiology (Vamathevan et al., 2019). As a result, their adoption in metabolomics has grown rapidly, driving innovation and progress. AI/ML approaches include K-Nearest Neighbors (K-NN) (Ehsani & Drabløs, 2020), Support Vector Machine (SVM) (Noble, 2006), Random Forest (RF) (Rigatti, 2017), Gradient Boosting (Natekin & Knoll, 2013), Least Absolute Shrinkage and Selection Operator Regression (LASSO Regression) (Fontanarosa & Dai, 2011), Artificial Neural Network (ANN) (Zou et al., 2008), Ensemble Learning (Mahajan et al., 2023), Convolutional Neural Network (CNN) (Alzubaidi et al., 2021), Alternating Decision Tree (ADTree) (Podgorelec et al., 2002), Multiple Linear Regression (MLR) (Sperandei, 2014), Partial Least Squares Discriminant Analysis (PLS-DA) (Gromski et al., 2015), Linear Discriminant Analysis (LDA) (Hu et al., 2020), and Logistic Regression (LR) (Zhang et al., 2021).

**Fig. 1:**
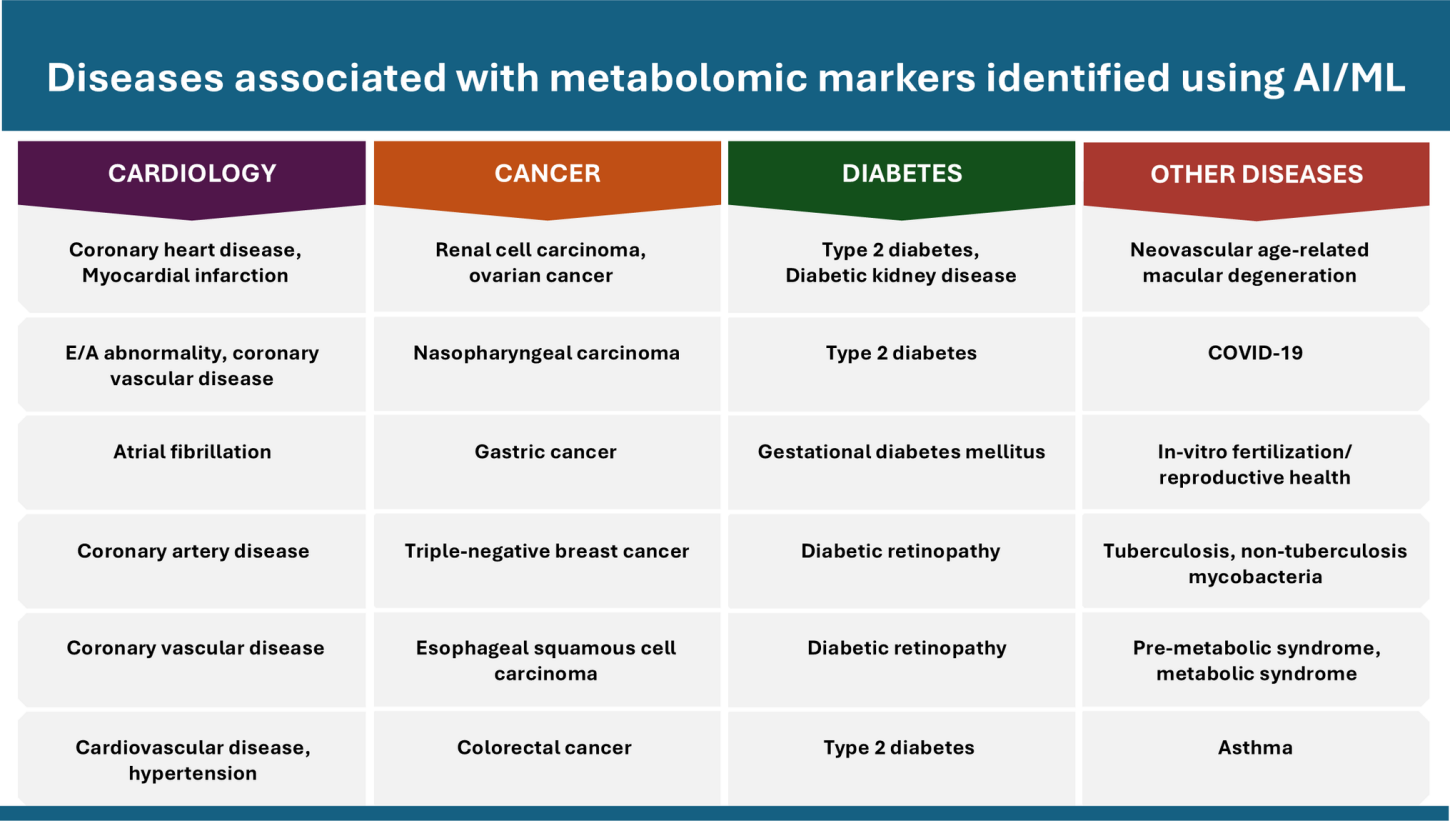
Disease overview. Overview of the diseases discussed in this study

In this study, we reviewed state-of-the-art literature focused on the application of AI/ML algorithms to metabolomic data for precision medicine. Our analysis targeted high-quality metabolomic studies published within the last five years (2019–2024) as indexed in PubMed. We specifically reviewed papers discussing the use of AI/ML techniques in conjunction with metabolomic data to predict or assess disease. We searched for AI/ML metabolomic data-based applications that would be user-friendly, replicable, interactive, intuitive, and would potentially integrate multi-omics data beyond just metabolomic data. Our search utilized keywords such as "Artificial Intelligence," "Machine Learning," "Metabolomics," "LC–MS," "Untargeted Metabolomics," and "Targeted Metabolomics." This effort identified 24 studies addressing a wide array of diseases including cancer, cardiovascular diseases, diabetes, and other diseases such as COVID-19, tuberculosis, and asthma (Fig. 1). We have provided a succinct overview of which diseases were explored, the techniques used to collect metabolomic data, AI/ML methods applied, statistical methods used, and the metabolites/biomarkers that were found for each of the 24 studies (Table 1). These studies provide valuable insights into the application of AI/ML methods on metabolomic data to enhance disease prediction and diagnosis, as well as the potential for future improvements.

**Table 1:** Comparative analysis of studies reviewed based on diseases, data types, AI/ML methods, statistical methods, and metabolites/biomarkers

Studies	Diseases	Data types	AI/ML methods	Statistical methods	Metabolites/biomarkers
Biomarkers differentiating coronary heart disease and Myocardial Infarction (Shen et al., 2024)	Cardiology: CHD, MI	LC–MS	KEGG Pathway	PCA, LASSO, PLS-DA	Fructose pathway, Alpha-linolenic acid, Mannose pathway
Predicting Cardiovascular Disease risk factors (Drouard et al., 2024)	Cardiology: E/A abnormality, CVD	Epigenetic DNA methylation Illumina Epic array, and High-throughput NMR	Semi-supervised learning and Unsupervised autoencoders	N/A	Lipids, Cholesterol concentrations, Branched-chain amino acids, Citrate, Lactate
Biomarkers identifying stroke in Atrial Fibrillation (Zhang et al., 2024)	Cardiology: AF, Stroke-related AF	LC–MS	RF and LR	Welch’s t- test, FC, P-value of Welch’s t-test, GO analysis	Arachidonic acid pathway, Sertogenic systems
Using TPOT to predict coronary artery disease (Orlenko et al., 2020)	Cardiology: CAD	NMR-derived metabolic profiles	TPOT, RFE, SE with LR Classifier, SE with Multinomial Naïve Bayes Classifier, SS and Bernoulli Naïve Bayes	N/A	Linoleic acid, Total saturated fatty acid, Omega-6 fatty acid and polyunsaturated fatty acid cumulative coefficient > 2%
Analyzing dietary metabolic signature to predict cardiovascular disease (Shah et al., 2023)	Cardiology: Coronary vascular disease	LC–MS	Elastic net regression, and Cox regression models	Canonical correlation	Choline/carnitine metabolism, Butyrobetaine, Trimethylamine-N-oxide Betaine, Alpha-glycerophsphocholine, Glutamate, Glutamine
Identifying metabolomic profiles to risk stratify cardiovascular disease patients (Moskaleva et al., 2022)	Cardiology: CVD, Hypertension	LC–MS	Neural Network, RF, SVM, LR, Bagging classifier, Gradient boosting	ANOVA	Trp, Methylarginine, Glycine, ADMA, Carnitine, Acylcarnitines, Kynurenine, Indole
AutoML-XAI: Advancing Metabolomics in Cancer Diagnostics (Bifarin & Fernández, 2024)	Cancer: RCC, OC	LC- MS	AutoML, K-NN, SVM, RF, Gradient, Boosting	SMOTE, SHAP	Dibutylamine, GM3 (d34:1)
AI-Driven Metabolic Mapping for Nasopharyngeal Carcinoma Detection (Xu et al., 2024)	Cancer: NPC	Single-Cell Raman spectroscopy, and UPLC-MS/MS	SVM	PCA, LDA	Glutamate, Unsaturated fatty acids, Nucleic acids
Metabolomics for Gastric Cancer Diagnosis and Prognosis (Chen et al., 2024)	Cancer: GC	LC–MS	RF, SVM, PLS-DA, LASSO	KEGG pathway enrichment analysis, PCA, LASSO	Succinate, Uridine, Lactate, ADMA, Neopterin
AI-Powered Metabolomics for Predicting Chemotherapy Response in Breast Cancer (Irajizad et al., 2022)	Cancer: TNBC	LC- MS	ANN, RF, Gradient boosting, Ensemble learning	N/A	Polyamines (Putrescine, spermidine, and spermine)
Transforming LC–MS Data into AI-Driven Diagnostic Images (Wang et al., 2023)	Cancer: ESCC	LC- MS	CNN	N/A	LPC (18:3), Carnitine (14:1)
Salivary Metabolomics for Colorectal Cancer Detection (Kuwabara et al., 2022)	Cancer: CRC	CE-MS, LC- MS	ADTree, MLR, PLS-DA	Agilent MassHunter Qualitative Analysis, Mann–Whitney test, PLS-DA	N-acetylputrescine, N-acetylspermine
ML for identifying kidney dysfunction associated metabolites in Type 2 Diabetes patients (An et al., 2024)	Diabetes: T2D, DKD	Demographic: SBP, DBP Clinical: SCR, cholesterol, triglycerides, HDL-C, LDL-C LC–MS	LR, SVM, RF, XGBoost	SHAP, OPLS-DA	C5DC, C10, eGFR
Integrated bioinformatics and tree-based ML techniques for metabolic biomarker discovery (Yagin et al., 2024)	Diabetes: T2D	LC–MS, UHPLC-MS	LightGBM, AdaBoost, XGBoost	PLS-DA, AUC, FDR, FC	AMP, ADP
ML and longitudinal metabolomics for biomarker discovery in Gestational Diabetes Mellitus (Lu et al., 2023)	Diabetes: GDM	HMDB, KEGG	SVR, LASSO	PCA, OPLS-DA, AUC, FC	Allantoic acid
Diagnosing Diabetic Retinopathy in T2D using XAI (Yagin et al., 2023)	Diabetes: NDR, NPDR, PDR	BMI, HbA1c, Glucose, Creatinine	mRMR, EBM, NGBoost, XGBoost	N/A	Trp, PC.aa.C42.2, C4, Tyr, C16, DMA
Metabolomic and proteomic biomarkers for Diabetic Kidney Disease (Liu et al., 2021a)	Diabetes: DKD	GC–MS	LDA, SVM, RF, LR, LASSO	PLS-DA	α2-M, CD324, CTSD, Glycerol-3-galactoside
ML for discovering T2D metabolomic biomarkers (Leiherer et al., 2024)	Diabetes: T2DM	LC–MS, FIA-MS	TreeBag, Caret, RF, SHAP, SVM, XGBoost	N/A	Hexoses, Glycine, Isoleucine, Kynurenine, CDCA, DCA
Biomarkers in Neovascular Age-related Macular Degeneration via iRF (Künzel et al., 2024)	Other Conditions: nAMD	LC–MS	Iterative RF	N/A	PFOS, Ethyl β-glucopyranoside
COVID-19 Metabolic Biomarkers Identified by ML (Elgedawy et al., 2024)	Other Conditions: COVID-19	UPLC-MS	RF	PLS-DA, OPLS-DA	Arginine, Malonyl methylmalonyl succinylcarnitine, and Tauroursodeoxycholic acid
Metabolomics Assay for Embryo Implantation Prediction (Cabello-Pinedo et al., 2024)	Other Conditions: IVF/Reproductive health	UHPLC-MS, OT-FTMS	GSEA, K-fold cross-validation, Fusion method	Mummichog	Tryptophan, Arginine, Proline, and Lysine
Biomarkers for Tuberculosis and Nontuberculous Mycobacteria via Metabolomics and ML (Anh et al., 2024)	Other Conditions: TB, NTM	LC–MS	RF, SVM, XGBoost, K-Nearest, Neighbors, Neural network	AUC	Methionine, Valine, Glutarate, 3-hydroxyanthranilate, Indole-3-carboxyaldehyde, and Corticosterone
ML and Lipidomics Reveal Pre-Metabolic and Metabolic Syndrome Biomarkers (Huang et al., 2024)	Other Conditions: Pre-MetS, MetS	UHPLC-MS	SVM, RFE, RF, LASSO, LDA	OPLS-DA	Phosphatidylserine, Diacylglycerol, and Triglycerides
CH25H Identified as Asthma Biomarker via ML (Ding et al., 2023)	Other Conditions: Asthma	LC–MS	LASSO, SVM, RFE, XGBoost, RF	Weighted Gene Co-Expression Analysis	CH25H, LPC (16:0), LPC (18:1), and LPA (18:1)

## Cardiology

### Biomarkers differentiating coronary heart disease and myocardial infarction (Shen et al., 2024)

Coronary heart disease is the most common type of heart disease in the United States, while myocardial infarction is a leading cause of death. This study performed by Shen et al. aimed to identify biomarkers and disrupted metabolic pathways unique to coronary heart disease (CHD) (Henderson, 1996) and myocardial infarction (MI) (Lu et al., 2015). By analyzing serum metabolic profiles, the research sought to distinguish between CHD and MI in a Chinese cohort using advanced ML and statistical models. Serum samples from 243 participants were collected, including 83 CHD patients, 73 MI patients, and 87 healthy controls. Metabolic profiling using LC–MS (Thomas et al., 2022) was conducted using an untargeted approach, yielding data on 702 metabolites across the three groups. Statistical models such as PCA (Groth et al., 2013), PLS-DA (Gromski et al., 2015), and LASSO (Fontanarosa & Dai, 2011) were used to explore the metabolic differences among the groups. PCA initially separated MI from controls, and PLS-DA provided further differentiation between CHD, MI, and controls. Univariate analysis identified metabolites with significant dysregulation, and KEGG pathway analysis (Kanehisa & Goto, 2000) highlighted the associated pathways. Finally, a LASSO regression model was developed to pinpoint specific biomarkers and assess their predictive performance using ROC curve analysis (Çorbacıoğlu & Aksel, 2023). The LASSO regression technique is generally employed in biochemical contexts for variable selection and for regularization in linear regression analysis, aiming to minimize the sum of squared residuals while aligning with the constraint of the sum of the absolute values of regression coefficients being equal or below a defined constant (Hong et al., 2024).

The study identified 80 dysregulated metabolites among the three groups. Pathway analysis revealed five major pathways affected, including alpha-linolenic acid metabolism for CHD and fructose, mannose, and glycolysis/gluconeogenesis pathways for MI (Shen et al., 2024). LASSO modeling identified three biomarkers (*acetyl carnitine*, *arginine*, *hypoxanthine*) that could differentiate MI from CHD, achieving an area under the curve score of 0.92 and 0.88 in training and test sets, respectively. An Area Under the Curve (AUC) score, specifically the AUC under the Receiver Operating Characteristic (ROC), used most often in ML and biochemical contexts, measures the ability of a given classifier to distinguish between true positives and false positives. A score of 1.0 represents perfect ability to distinguish between the two classes (diseased vs. healthy) and a score less than 0.5 represents a classification ability that is not better than a random classification (Çorbacıoğlu & Aksel, 2023). CHD and MI were further distinguished from controls using distinct sets of metabolites with AUCs around 0.8–0.9 (Shen et al., 2024). This approach of combining untargeted metabolomics with ML demonstrates potential for precise and early differentiation of CHD and MI (Shen et al., 2024). This also implies that untargeted metabolomics, when analyzed by AI/ML, can reveal new information in well-studied diseases (Shen et al., 2024). Future studies may need to utilize a larger, more diverse, multi-cohort sample to allow for greater generalizability of study findings and broader clinical application. Additionally, the study did not incorporate long-term patient outcomes, limiting insights into the prognostic value of the identified biomarkers.

### Predicting cardiovascular disease risk factors (Drouard et al., 2024)

Cardiovascular disease (CVD) encompasses coronary heart disease, heart failure, atrial fibrillation, and more, affecting millions of people in the United States, so to improve outcomes and prevent hospitalization, it is important to find early indicators of disease manifestation. This study investigated various ML strategies for predicting CVD (Goldsborough et al., 2022) risk factors using multi-omics data under different scenarios. These scenarios included assessing the impact of different ML classifiers, omics types, and dimension reduction techniques on prediction quality. The researchers examined model interpretability in semi-supervised autoencoders to identify which omics factors contributed most to dimension reduction. They also investigated scenarios in which late-integrative multi-omics modeling outperformed single-omics modeling and explored whether transfer of omics representations acquired by semi-supervised autoencoders could improve CVD risk factor prediction in an external cohort. Blood-derived omics information such as transcriptomic, metabolomic, and epigenetic data were collected from 1,650 individuals in the Young Finns study (Raitakari et al., 2008). Illumina EPIC array was used to quantify epigenetic DNA methylation. Illumina microarray technology was used for transcriptomic data whereas high-throughput NMR was used for metabolomic data. Only 1,249 participants qualified because omics data and CVD biomarkers overlapped. The YFS participants were randomly divided into a training set (n = 1,000; ~ 80%) and an independent test set (n = 249; ~ 20%). Within the training set, fivefold cross-validation was employed for model selection and hyperparameter tuning. All preprocessing procedures, including scaling and feature filtering, were performed exclusively on the training data, and the derived parameters were applied to the test set to minimize the risk of information leakage. Nevertheless, because biomarker-based 1-standard deviation classes were defined relative to cohort means, some overlap in population-level distributions may have introduced indirect leakage. This limitation should be considered when interpreting predictive performance. For data processing, the researchers used six ML classifiers to predict risk factors like systolic and diastolic blood pressure as well as biomarkers for left ventricular diastolic dysfunction. To set up dimension reduction, the researchers used semi-supervised (Qin et al., 2022) and unsupervised autoencoders (Baur et al., 2021). These models were trained to simultaneously reconstruct the original omics inputs from corrupted data and predict cardiovascular biomarker values. The bottleneck layer provided latent features that summarized omics variation while enhancing predictive relevance. These compressed features were subsequently used as inputs for downstream classifiers. Variable importance within the SSAE was assessed using the Connection Weights algorithm, which quantified the contribution of individual metabolites and transcripts to the reduced representations. The model’s prediction quality by the F1 score (a performance metric that represents harmonic mean by balancing precision and recall), which specializes in imbalanced designs where most individuals have moderate risk levels (more diseased rather than healthy individuals). An F1 score is the harmonic meaning of precision and recall scores of the number of true positives that the model detects, with scores of 1.0 representing perfect precision and recall and a score of 0 representing no overlap between predicted positives and actual positives (Hicks et al., 2022). Finally, they tested transfer learning by using pre-trained autoencoders and observed if predictions improved with a different task in an external cohort (Finnish Twin Cohort) where a higher proportion of individuals were hypertensive.

The researchers found that multi-omics predictions generally outperformed single-omics with better macro-F1 scores for individuals with high or low levels of risk, but there were still scenarios where single-omics performed better such as for systolic blood pressure (SBP) (Basile, 2002) and E/A ratio, a measure of the heart’s left ventricle function (Wu et al., 2023) with metabolomics (macro-F1 of 0.51 vs. 0.50 for multi-omics) and transcriptomic data (macro-F1 of 0.44 vs 0.38) respectively (Drouard et al., 2024). Multi-omics predictions outperformed single-omics predictive modeling in predicting 1-sd classes of blood pressure 83% of the time. Furthermore, the semi-supervised autoencoder models yielded better prediction rates compared to unsupervised models. For example, the semi-supervised transcriptomic models improved predictions of individuals deviating more than 1SD from the mean in 73% for semi-supervised and 60% for unsupervised (Drouard et al., 2024). For metabolomic predictions, macro-F1 predictions from semi-supervised models outperformed unsupervised models half the time. With semi-supervised encoding, they found that the metabolomic features of highest absolute variable importance in the reconstructions of Left Ventricular Diastolic Dysfunction (LVDD) biomarkers were lipids, cholesterol concentrations, lactate and citrate. With multi-omics approaches, it is difficult to externalize the models to other cohorts, but the researchers found transfer learning successful when they saw notable gains in area under the curve (AUC) from 62.7% to 72.9% with transfer learning alone and from 64.9% to 69.4% from increasing the fine-tuning sample size from 20 to 80% for metabolomic and transcriptomic data in predicting CVD risk factors in the external cohort (Drouard et al., 2024). They saw a greater AUC in the transfer-based model in the external cohort than in the built-from-scratch models. The study highlights the potential of using multi-omics approaches to improve the prediction of CVD risk factors, but costs and benefits still need to be considered when adding omics layers because it increases model complexity in the training set, which could also reduce model reproducibility in external cohorts. The study also suggests that the predictive potential of metabolomic data compared to other omics varies depending on which ML classifiers are used, such as RF or gradient boosting machine (Drouard et al., 2024). The study confirms that methods specifically designed for multi-omics data integration are beneficial, as demonstrated in semi-supervised learning and transfer learning. To improve the study, a larger cohort could have been used to train the model, which would allow it to be used for a more generalizable population. Additionally, due to the sample population being relatively healthy, it was more difficult to identify individuals with a substantial CVD risk.

### Biomarkers identifying stroke in atrial fibrillation (Zhang et al., 2024)

Patients with atrial fibrillation (AF) have a higher risk of stroke due to the irregular heart beating caused by AF, causing susceptibility of blood clot formation, which can travel to the brain. By finding early indicators, care teams could better prevent strokes in AF patients. In this study, Zhang et al., sought to identify proteomic and metabolomic signatures of stroke in AF (Sagris et al., 2021) patients to better understand the molecular mechanisms behind stroke in AF (Elsheikh et al., 2024) and develop predictive biomarkers using the RF method. Plasma samples were collected from 55 AF patients including 30 patients without stroke and 25 with ischemic stroke, sourced from Harbin Medical University in China. Samples were analyzed using (LC–MS/MS) (Thomas et al., 2022) for proteomic and metabolomic profiling. Differentially expressed metabolites (DEMs) were identified using p-value of Welch’s t-test (West, 2021) and variable importance in the projection of partial least squares discriminant analysis, as well as FC (Wiebe et al., 2020) and variable importance in the projection of partial least squares discriminant analysis. The researchers used spearman correlation analysis to identify the correlation between DEPs and DEMs. To screen a combination of proteomics and metabolomics biomarkers, the researchers developed a RF model (Rigatti, 2017). Then, they used logistic regression (Sperandei, 2014) to correct the impact of imbalance clinical parameters on potential biomarkers. KEGG (Kanehisa & Goto, 2000) and GO (Ashburner et al., 2000) analyses were conducted to investigate protein and metabolite pathways such as arachidonic acid metabolism, serotonergic synapse, and ovarian steroidogenesis. A RF classifier was used to predict stroke in AF patients.

The researchers collected proteomic data, identifying 1798 proteins in plasma samples with 53 DEPs. Metabolomic data was also collected with 2,303 metabolites identified with 114 DEMs. 6 proteomic derived biomarkers (*CLEC3B*, *C4B*, *IGHV1-58*, *LGALS1*, *NME1,* and *VH6DJ*) and 6 metabolomic derived biomarkers (*CLEC3B*, *N-benzylfromamide*, *Glycitin*, *Tiapride*, *6-Methyltionosine*, *Prostaglandin H1*, and *Prosglandin F2alphamethyl ester*) were identified as potential biomarkers for predicting stroke in AF patients (Zhang et al., 2024). Using a tenfold cross validation of RF, the candidate biomarkers had a high accuracy in predicting stroke at an AUC of 0.976, which was significantly higher than D-Dimer, a protein fragment in the blood that indicates whether a blood clot is present (the current measure used for prediction). KEGG analysis revealed several significantly enriched pathways, including arachidonic acid metabolism, serotonergic synapse, and steroid hormone biosynthesis. By using a multi-omics approach, the authors provide a novel method for understanding AF-induced stroke (Zhang et al., 2024). This study shows that larger datasets can be used to yield better biomarker candidates (Zhang et al., 2024). Due to the small sample size of 55 patients and no external cohort usage to validate findings, future studies may need to utilize a larger sample size and an external cohort to be able to further validate findings.

### Using TPOT to predict coronary artery disease (Orlenko et al., 2020)

Coronary artery disease (CAD) reduces blood flow to the heart due to plaque buildup. It is the most common type of heart disease in the United States. In this study, Orlenko et al., aimed to predict CAD diagnosis (Malakar et al., 2019) using metabolomic data and AutoML. The primary goal was to evaluate the performance of various ML models, including those optimized by the tree-based pipeline optimization tool (TPOT) (Le et al., 2020) in identifying CAD phenotypes. The dataset comprised nuclear magnetic resonance (NMR)-derived metabolite profiles (Cerulli et al., 2021), including 73 metabolic markers, and 27 demographic and clinical features from the ANGES cohort of 925 patients undergoing coronary angiography. Patients were categorized into three groups: functionally obstructive CAD stenosis, non-obstructive CAD, and no CAD using angiographic findings. For ML analysis, these groups were recoded into binary outcomes to reflect clinically relevant comparisons—Profile 1 (P1): no CAD versus combined non-obstructive and obstructive CAD and Profile 2 (P2): combined no CAD and non-obstructive CAD versus obstructive CAD. These profiles align with clinical decision-making, where the distinction between obstructive and non-obstructive CAD reflects treatment thresholds, while the separation of CAD (obstructive or not) from no CAD captures overall disease presence (Orlenko et al., 2020).

The dataset was split into 75% training and 25% validation. The TPOT AutoML tool was applied to generate ML pipelines for predicting CAD diagnoses, comparing obstructive, non-obstructive, and no CAD groups. Four models were run for each phenotypic profile for 1000 generations, spanning 24 h with a population size of 1000 pipelines. The first model was TPOT with full configuration, with a complete list of data operators and ML classification models. The Second Model was a reduced configuration with logistic regression classifier and a complete list of data transformers and selectors. The third Model was a reduced configuration with decision tree classifier and a complete list of data transformers and selectors. The last Model was a reduced configuration with RF classifier and a complete list of data transformers and selectors. They compared TPOT-based model selection to the exhaustive grid search parameter using various ML models (logistic regression (Sperandei, 2014), decision trees, random forest (Rigatti, 2017), and Bernoulli Naïve Bayes (BNB) (Baur et al., 2021)) to maximize performance metrics like accuracy and precision.

The TPOT-optimized models outperformed traditional grid search approaches, with the best-performing model achieving a balanced accuracy of 0.78 for predicting CAD. Full configuration pipeline Model A1 had accuracy 0.77 containing four pre-processing operations (RFE, SE with LR classifier, SE with Multinomial Naïve Bayes Classifier, SS and BNB as ML classifier) for the first phenotype. For the second phenotype, TPOT with full configuration produced a pipeline with four pre-processors (Variance Threshold, RFE, SE with LR, Max Abs Scaler) and BNB with a balanced accuracy of 0.78 (Orlenko et al., 2020). The study also identified significant clinical and metabolic features, such as HDL cholesterol and fatty acids, as the most important features that contributed to CAD diagnosis predictions. The study demonstrated that AutoML can effectively select and optimize ML pipelines for clinical applications, offering a valuable approach for predicting CAD using metabolic and clinical data (Orlenko et al., 2020). These findings have the potential to help improve the precision of CAD diagnostics and risk stratification in clinical practice. Areas for improvement include that the study was limited to the ANGES cohort and the model's generalizability to other populations remains untested. The use of AutoML is also significantly resource-intensive, particularly when evaluating large datasets with numerous features and model configurations, which can create problems for replicability and practical applications of this method. Regarding computational resources, running 1000 TPOT pipelines for 24 h requires hardware beyond a standard commercial laptop. In the ANGES study, optimization was performed on a high-memory desktop environment with multicore processors.

### Analyzing dietary metabolic signature to predict cardiovascular disease (Shah et al., 2023)

In this study, Shah et al., aimed to investigate the association between dietary patterns and long-term cardiometabolic-cardiovascular disease (CM-CVD) (Goldsborough et al., 2022) using metabolic signatures. The goal was to understand how analyzing specific metabolite profiles, derived from food intake, can better predict CM-CVD outcomes compared to traditional dietary assessments. The study utilized metabolomic data collected via LC–MS from over 2,259 participants in the CARDIA cohort and 2,006 participants from the Framingham Heart Study (FHS). Metabolite profiles included amino acids, lipids, carbohydrates, and other metabolites, alongside detailed dietary intake information and clinical outcomes related to diabetes and CVD. The researchers employed ML techniques, including elastic net regression (Malakar et al., 2019) and regularized sparse canonical correlation analysis (CCA) (Yoon et al., 2020), to identify metabolite signatures associated with 17 food groups and dietary patterns. Cox regression models (Zhang et al., 2018) were used to assess the association between these metabolite signatures and long-term risks of diabetes and CVD, adjusting for demographic, clinical, and lifestyle covariates. The study found that elastic net regression (Münch et al., 2021), CCA, and Cox regression model applications to metabolite profiles significantly improved the prediction of long-term diabetes and CVD risk compared to traditional dietary assessments and standard of care. In the CARDIA cohort, the addition of metabolites to models of dietary intake increased the predictive accuracy, particularly for food groups like red meat and sugary drinks. Over a 23-year follow-up, metabolite-based dietary scores were strongly associated with diabetes (HR: 1.62, 95% CI 1.32–1.97) and CVD (HR: 1.55, 95% CI 1.12–2.14), outperforming traditional Healthy Eating Index scores. Similar results were replicated in the Framingham Heart Study, where the metabolite scores were significantly associated with diabetes risk (HR: 1.38, 95% CI 1.20–1.59) over 18 years (Shah et al., 2023). These findings highlight the value of metabolomics in enhancing diet-related predictions of cardiometabolic outcomes (Shah et al., 2023). These findings also suggest that metabolite-based dietary signatures detected by ML methods can offer more accurate insights into the long-term health effects of diet, which could influence personalized dietary recommendations and preventive strategies for CM-CVD (Shah et al., 2023). The authors stated that this study relied on self-reported dietary data for assessment, which may introduce bias and skewed results. In addition, metabolite changes were only sourced from chronic dietary intake, but transient metabolite changes sourced from acute food consumption, which was not accounted for, could also impact results. Future studies would also need to integrate greater geographical and racial diversity to be able to fully generalize findings.

### Identifying metabolomic profiles to risk stratify cardiovascular disease patients (Moskaleva et al., 2022)

Cardiovascular disease is a leading cause of death in the United States, and conditions such as hypertension (high blood pressure) are major risk factors. In this study, Moskaleva et al., aimed to evaluate the use of targeted metabolomics and ML for risk stratification of patients with coronary artery disease (CAD) (Malakar et al., 2019), hypertension (HTA) and non-CVD individuals. Non-CVD patients were included as a control group to the CAD cohort. The study used plasma samples from 136 adults: 61 with HTA, 48 with CAD, and 27 with no CVD. Participants were recruited in Moscow, Russia between 2018 – 2020. Metabolites analyzed included amino acids, acylcarnitines, and compounds from the tryptophan metabolic pathway (Wyant & Moslehi, 2022), assessed via liquid chromatography-tandem mass spectrometry (Thomas et al., 2022). The Shapiro–Wilk test was utilized to check distribution of variables. As the majority of metabolites did not follow a normal distribution, non-parametric methods (Kruskal–Wallis with Benjamini–Hochberg false discovery rate correction) were applied for group comparisons. Parametric ANOVA was performed only for variables that satisfied normality. Features with a corrected *q* value < 0.05 were considered significant. AUCs comparing the non-CVD group with the CVD group were used for diagnostic accuracy. For multiple classification, ML models including RF (Rigatti, 2017), gradient boosting (Natekin & Knoll, 2013), neural networks (Zou et al., 2008), and SVM (Valkenborg et al., 2023), were applied to classify patients with HTA, CAD, and non-CVD. For binary classification, logistic regression (Sperandei, 2014), RF classifier, multiple neural networks, gradient boosting, support vector classifier, and bagging classifier (Zhang et al., 2021) were used. Models were evaluated for accuracy using AUC and F1 scores. Accuracy scores helped identify the best approach for predicting CVD status based on metabolite data.

The RF model (Rigatti, 2017) achieved the highest predictive accuracy (80% for multiclass classification and 91% for binary CVD vs. non-CVD classification). The AUC was 0.91 and the F1 score was 0.90. The AUCs for gradient boosting classifier was 0.86, MLP classifier was 0.81, support vector classifier was 0.78, logistic regression was 0.71, and bagging classifier was 0.58. Using heatmap correlation matrices, researchers saw that *LVEF*, *LDL-C*, *HDL-C* and *uric acid* were the most consistent cardiometabolic parameters to be associated with analyzed metabolites. Using univariate analysis and BH-FDR correction, researchers found that the key diagnostic metabolites included elevated kynurenine/tryptophan ratios and certain acylcarnitine*s,* suggesting inflammation and metabolic shifts as CVD indicators (Moskaleva et al., 2022). Intermediates of the tryptophan pathway metabolites such as *Indole-3-butyric acid, serotonin, and kynuric acid* were helpful in distinguishing CVD stages because tryptophan is involved in serotonin production, kynureine, and indole pathways. Elevated kynurenine/tryptophan ratios and increased levels of downstream kynurenine pathway intermediates (kynurenic acid, quinolinic acid, anthranilic acid) were observed alongside higher prevalence of hypertension within the CVD cohort. This interaction suggests that chronic blood pressure elevation may accelerate tryptophan degradation through inflammatory and oxidative stress pathways, consistent with prior reports linking hypertension to altered kynurenine metabolism (Bartosiewicz et al., 2017). Based on the predictive accuracies, the RF model provided the best predictive power for the stratification of early CVD patients based on target metabolomic profiling, demonstrating the RF model’s potential applications in future experiments and diagnostic inquiries related to CVD (Moskaleva et al., 2022). However, the authors report a small sample size and a lack of external validation, which should be considered in generalizing the study findings. Additionally, the study's cross-sectional design could not establish causative relationships between metabolites and disease progression, because a cross-sectional study takes patient data from one point in time rather than following them over a longer period that would enable more evidence for a causative relationship.

## Cancer

### AutoML-XAI: advancing metabolomics in cancer diagnostics (Bifarin & Fernández, 2024)

This study aimed to compare AutoML techniques with traditional ML algorithms, such as SVM (Noble, 2006), RF (Rigatti, 2017), and K-NN (Ehsani & Drabløs, 2020), in the analysis of metabolomic data derived from LC–MS (Bifarin & Fernández, 2024; Seger & Salzmann, 2020). AutoML (Automated Machine Learning) refers to the process of automating the end-to-end process of applying ML to real-world problems, including data preprocessing, model selection, hyperparameter tuning, and deployment, making it accessible to non-experts and improving efficiency. The researchers’ goal was to determine whether AutoML can provide an effective and accessible solution for non-experts in ML. The Renal Cell Carcinoma (RCC) dataset consisted of 82 RCC patients and 174 healthy controls, while the Ovarian Cancer (OC) dataset included 208 Korean women with OC and 117 women with other gynecological malignancies (non-OC). They collected two metabolite discriminant panels: a 7-urine metabolite panel for detecting RCC and a 17-serum lipid panel for detecting human OC. Both datasets were generated using LC–MS analysis. The authors used Auto-Sklearn as the AutoML tool of choice, which automates the selection of ML pipelines through Bayesian optimization, meta-learning, and ensemble construction. For the RCC data, they split the data 80:20 for training and testing respectively, while the OC data was split 70:30. The OC training set was further balanced using the Synthetic Minority Oversampling Technique (SMOTE) to address the class imbalance. The authors compared the performance of AutoML with standalone traditional algorithms such as RF, SVM, and K-NN. They allocated 600 s for Auto-Sklearn to build the best ML pipeline for the RCC data and 3600 s for the OC data. After the AutoML pipeline was constructed, they performed a fivefold cross-validation (CV) and tested the models on unseen datasets alongside the traditional algorithms.

For the RCC dataset, Auto-Sklearn’s final ensemble model consisted of 13 algorithms, including SVM, gradient boosting, and the extra_trees algorithm. The ensemble achieved an AUC score of 0.97, surpassing RF (0.95) and matching SVM and RF in accuracy (0.90) (Bifarin & Fernández, 2024). Auto-Sklearn also outperformed both RF and SVM in sensitivity (0.89 vs. 0.83). However, RF and SVM had better specificity scores (0.94 vs. 0.91 for Auto-Sklearn). K-NN consistently had the lowest scores across all metrics. In the OC dataset, Auto-Sklearn outperformed traditional algorithms, with an AUC of 0.85 (matching RF) and achieving the highest accuracy (0.78) and specificity (0.82). It also matched RF in sensitivity (0.75). K-NN had the lowest performance metrics across the board. The authors used Shapley Additive Explanations (SHAP) to identify the most important metabolites for disease prediction*.* SHAP is an approach that allows both prioritization and identification of features that determine classification and prediction using any ML model (Rodríguez-Pérez & Bajorath, 2020). *Dibutylamine* had the most significant impact on RCC prediction, while *GM3* (d34:1) was the most important metabolite for distinguishing OC patients from non-OC patients. This study demonstrates the potential of AutoML to automate complex ML tasks, providing results that match or exceed those of traditional ML algorithms. The ability of AutoML to optimize model pipelines with minimal human intervention makes it a valuable tool for non-experts working in metabolomics (Bifarin & Fernández, 2024). Although AutoML showed superior performance in most cases, the authors note that further validation is needed on more diverse datasets. Additionally, comparing other AutoML platforms or machine learning algorithms could provide further insight into the robustness and generalizability of these results.

### AI-driven metabolic mapping for nasopharyngeal carcinoma detection (Xu et al., 2024)

Nasopharyngeal carcinoma (NPC), a malignant tumor of the nasopharyngeal cavity, is often diagnosed at advanced stages due to its subtle, early symptoms and lack of reliable early biomarkers. Addressing this critical limitation, the study aimed to identify reliable biomarkers for the early detection of NPC data processed using single-cell Raman spectroscopy (Li et al., 2012) through a SVM approach (Noble, 2006; Xu et al., 2024). The study involved seven NPC cell lines (5-F, 6-10B, SUNE-1, CNE1, CNE2, C666-1, and HK1) and one non-NPC epithelial cell line (BMI1). Ultra-Performance Liquid Chromatography-Tandem Mass Spectrometry (UPLC-MS/MS) (Fernández & Samyn, 2011) was employed for metabolite identification by comparing ion features to an in-house reference library (Xu et al., 2024) of the 13 patient tissue samples used, 7 were from NPC tissues, and 6 were from nasopharyngeal mucosa with inflammation, collected from 12 men and 1 woman. The authors utilized single-cell Raman spectroscopy, which enables real-time chemical analysis of cells. ML models were applied to these eight cell lines. The authors visualized single-cell Raman spectral data using Linear Discriminant Analysis (LDA) to distinguish Raman groups. The dataset was split 70:30 into training and testing sets, and Principal Component Analysis (PCA) reduced the data to the 100 most significant wavenumbers, preserving 94.5% of the variance. The training data was then used to train an SVM model with tenfold cross-validation. Additionally, UPLC-MS/MS was employed for metabolite identification by comparing ion features to an in-house reference library.

The authors observed significant differences in intracellular macromolecule content between NPC and non-NPC cells. NPC cells exhibited increased nucleic acid content, while protein levels varied across NPC lines, with some showing increases and others decreases compared to the non-NPC cell line. Saturated lipid levels decreased in all NPC lines, though unsaturated lipid content varied. Glucose levels were inconsistent among NPC cells, while all NPC lines showed reduced *glutamate* levels. Notably, the highly metastatic 5-8F NPC cells had higher unsaturated fatty acid content compared to the less metastatic 6-10B cells, which was confirmed by UPLC-MS/MS data. Using the SVM-PCA model to distinguish between NPC and non-NPC cells, the authors achieved an area under the curve (AUC) of 0.99, with 93% sensitivity and 99.2% specificity (Xu et al., 2024). The model correctly classified test data into eight groups with an accuracy of 85.5%, and the non-NPC cells showed 99% specificity, minimizing the likelihood of false positives. When tested on 13 patient tissue samples (7 NPC, 6 non-NPC), the model accurately predicted all cases, achieving an AUC of 0.97. This study demonstrates that metabolic changes can help understand cancer progression and metastasis (Xu et al., 2024). The high diagnostic accuracy of the SVM model in distinguishing between NPC and non-NPC cells suggests that this approach holds promise for early detection, with minimal risk of false-negative results (Xu et al., 2024). The authors acknowledge the need for further validation using larger datasets and more tissue samples. Additionally, exploring other ML algorithms could provide a performance benchmark against the SVM model used in this study.

### Metabolomics for gastric cancer diagnosis and prognosis (Chen et al., 2024)

The goal of this study was to identify non-invasive biomarkers for early detection of gastric cancer and assess patient prognosis. This study utilized 702 plasma samples from a multi-center cohort, including 389 gastric cancer (GC) patients and 313 non-gastric cancer (NGC) patients (Chen et al., 2024). Targeted liquid metabolomics analysis was conducted using LC–MS (Thomas et al., 2022), identifying 147 metabolites. The authors developed a 10-metabolite diagnostic model (10-DM) to differentiate between GC and NGC patients. To develop this model, they started by using principal component analysis (PCA) to identify 45 metabolites that were significantly varied between GC and NGC patients. KEGG pathway enrichment analysis was used to reveal disrupted metabolic pathways in GC patients (Chen et al., 2024). From there, the LASSO regression algorithm (Fontanarosa & Dai, 2011) identified the 10 most essential metabolites for discrimination between GC and NGC. A RF model (Rigatti, 2017) was then trained on these 10 metabolites to create the final 10-DM model. Each of the 10 metabolites contributed relatively equally to the model. The authors compared the performance of the 10-DM model with and without the inclusion of 3 existing tumor biomarkers and against other ML algorithms, including SVM (Noble, 2006), Logistic Regression (LR) (Sperandei, 2014), RF (Rigatti, 2017), and Partial Least Squares Discriminant Analysis (PLS-DA) (Chen et al., 2024; Gromski et al., 2015). Additionally, a 28-metabolite prognostic model (28-PM) was generated using data from 181 GC patients with a median follow-up of 40 months. A RF survival method was applied to create the model, which was reduced from 147 to 28 metabolites to prevent overfitting. Only 11 of these 28 metabolites significantly predicted survival in the test set (Chen et al., 2024). Clinical parameters were incorporated into the 28-PM model to assess its impact on performance.

The 10-DM model achieved an AUC of 0.967, sensitivity of 0.854, and specificity of 0.926 when validated with test set 1, an internal validation set split from the initial cohort (Chen et al., 2024). While the metabolites contributed relatively equally, *succinate, uridine, and lactate* were identified as the most significant metabolite contributors (Chen et al., 2024). The model correctly identified 85.4% of GC patients in test 1 and 90.3% in test set 2, an external independent cohort used for further validation (Chen et al., 2024). It also achieved a prediction accuracy of 90.9% for stage IA GC and 95% for stage IB GC patients. When tested with stage IA patients from test set 2, the model demonstrated an AUC of 0.92, sensitivity of 0.905, specificity of 0.75, and detection accuracy of 79.1%. When compared to a 3-biomarker panel (CA19-9, CA72-4, and CEA), the 10-DM model showed superior performance with a sensitivity of 0.925 versus 0.428. The model’s sensitivity increased to 0.957 when integrated with the 3-biomarker panel. The 10-DM model outperformed other ML algorithms like SVM, LR, RF, and PLS-DA. The 28-PM model identified 11 metabolites that significantly predicted overall survival (Chen et al., 2024). The model achieved an AUC of 0.832, sensitivity of 0.900, and specificity of 0.700 in the test set. Notable metabolites included *ADMA*, known to promote gastric cancer metastasis, and *neopterin*, which is associated with immune activation and prognosis in other cancers. The 28-PM model was compared to clinical factors (TNM staging, macroscopic appearance, and vascular tumor embolus), which were identified using univariate Cox regression analysis. The C-index showed the 28-PM model performed better than clinical factors alone. Incorporating clinical factors into the 28-PM model did not significantly improve its performance (Chen et al., 2024). The model was able to successfully categorize patients into high- and low-risk groups based on prognosis. This study demonstrated that ML models based on metabolomic data can accurately predict both diagnosis and prognosis in gastric cancer and outperform traditional clinical methods (Chen et al., 2024). The findings suggest that metabolites can effectively differentiate between GC and NGC (Chen et al., 2024). The cohort used to train the 28-PM model had a relatively short median follow-up time of 40 months, which should have been extended to more than 5 years (Chen et al., 2024). Additionally, the sample size was small and thereby reduced the potential for proper data splitting for testing and validation. The authors noted that their models are not yet ready for clinical application.

### AI-powered metabolomics for predicting chemotherapy response in breast cancer (Irajizad et al., 2022)

This study aimed to investigate whether metabolomics data can predict response to neoadjuvant chemotherapy (NACT), which is chemotherapy given before the main treatment, in patients with triple-negative breast cancer (TNBC) (Irajizad et al., 2022). The sample cohort consisted of 88 patients with TNBC with a mean age of 50, alongside a control group of 167 individuals with a mean age of 58 (Irajizad et al., 2022). To address potential pre-analytical factors affecting metabolite stability, the authors employed rigorous quality control measures, including normalization to quality control injections every 10 samples, applying Locally Weighted Scatterplot Smoothing (LOESS) signal correction to reduce signal fluctuations, and reporting values relative to historical QC medians, which enhance data reliability (Irajizad et al., 2022). A metabolite panel was used to measure two polyamines and nine other metabolites, with data collected using liquid chromatography-tandem mass spectrometry pre-treatment (Thomas et al., 2022). The TNBC patients underwent four cycles of Adriamycin-cyclophosphamide (AC) chemotherapy. A deep learning algorithm (Zou et al., 2008) was developed using hyperparameters optimized through a grid search method, repeated 20 times. The significance of each metabolite was evaluated using the Gedeon Method (Irajizad et al., 2022). The deep learning model was compared against RF (Rigatti, 2017), ensemble learning (Mahajan et al., 2023), and gradient boosting algorithms (Natekin & Knoll, 2013) for distinguishing chemotherapy responders from non-responders. The models were based on the 11 most important metabolites identified. A fivefold cross-validation (CV) was used to evaluate the models on the training set. Proper data splitting in CV is essential for validating a model's performance on new, unseen data. This is especially true for small datasets, which increase the risk of overfitting, where the model learns the training data on a small, specific dataset, and thereby does not generalize well to new data. The resulting top-performing deep learning model comprised three hidden layers with 20 nodes each; a configuration identified through hyperparameter tuning that is well-suited for a small sample size to mitigate the risk of overfitting. (Irajizad et al., 2022). It achieved an AUC of 0.97, with 85% sensitivity and 95% specificity in distinguishing responders from non-responders. This model outperformed the best gradient boosting algorithm (AUC of 0.61) and the best RF algorithm (AUC of 0.55). When tested on a subset of plasma samples from 62 TNBC patients, the deep learning model yielded an AUC of 0.74, 21% sensitivity, and 95% specificity. This notable decrease in performance from the cross-validation AUC of 0.97 to 0.74 on this subsequent dataset is a significant finding that may suggest model overfitting. It is important to clarify, however, that this second set of samples was collected mid-treatment (after four cycles of chemotherapy), not pre-treatment like the initial training data (Irajizad et al., 2022). To specifically assess for potential overfitting, the authors tested the model’s stability by introducing perturbations to the dataset and re-evaluating its performance, concluding that their model was robust (Irajizad et al., 2022). The authors demonstrated that their deep learning model could predict the distinction between responders and non-responders to NACT using blood-based metabolomics data, outperforming other ML approaches. This finding suggests the potential of deep learning models as a promising approach compared to other machine learning methods in this context for leveraging metabolomic data to predict and personalize diagnosis and treatment outcomes for TNBC patients (Irajizad et al., 2022). The study had a relatively small sample size and lacked external validation, which can cause problems for generalization and application of findings. The authors also pointed out that using other clinical predictors could be more cost-effective than using a metabolite panel (Irajizad et al., 2022).

### Transforming LC–MS data into AI-driven diagnostic images (Wang et al., 2023)

The study aimed to develop an AI-based model to differentiate between esophageal squamous cell carcinoma (ESCC) screening-positive (SP) and screening-negative (SN) subjects using serum metabolomics data. Additionally, the authors sought to retain the characteristics of the mass spectra in the encoded images to improve interpretability, which previous methods failed to do.A clinical cohort of 1,104 participants, all diagnosed with esophagitis, low-grade dysplasia (LGD), high-grade dysplasia (HGD), or ESCC was utilized. All participants provided a 5 mL peripheral venous fasted blood sample, which was prepared and used for Liquid Chromatography-tandem Mass Spectrometry (LC–MS) (Thomas et al., 2022) untargeted metabolomics analysis. The authors employed a Convolutional Neural Network (CNN) (Alzubaidi et al., 2021) as it had demonstrated high accuracy for clinical image-based disease diagnosis in previous studies performed. They transformed their untargeted LC–MS metabolomics data into images and input them into a CNN. Past methods often resulted in images that lost interpretability, turning deep learning models into “black boxes”. To address this, the authors introduced a new model called MetImage, which encoded the data into multi-channel images while preserving the characteristics of the raw LC–MS data. Specifically, the LC–MS data is first converted into a large 2D digital image representing the whole metabolome profile, with m/z values binned (0.01 Da width) along one dimension, retention time along the other, and ion intensities as pixel values. This matrix is then split into smaller 224 × 224-pixel tiles, which are stacked as a multi-channel image. By expanding the channels in this way, MetImage enhanced compatibility with deep learning frameworks while preserving key features of the mass spectra in each tile, maintaining full data resolution without compression loss. The raw LC–MS dataset contained a total of 6,617 image channels, from which 661 were selected to form multi-channel images. This selection was based on the intersection of the top 1,000 channels ranked by pooled intensity (PI) and entropy (H). The cohort of 1,104 participants was split into a training set of 458 participants, a validation set of 204, and a testing set of 442. The training set, comprising 458 multi-channel images (212 SN, 246 SP), was fed into a ResNet-18 model with 17 convolutional layers and one fully connected linear layer, each employing batch normalization. The model had an initial learning rate of 0.001 (Wang et al., 2023). The output of the model was a risk score between 0 and 1, where scores above 0.5 were classified as SP, and scores below 0.5 as SN.

The model achieved an AUC of 0.95, a sensitivity of 85%, and a specificity of 92%. It successfully distinguished between SP and SN with an overall accuracy of 90%. The model also demonstrated reliability with a Positive Predictive Value (PPV) of 87% and a Negative Predictive Value (NPV) of 91%. The authors adjusted the model to use a cut-off of 0.4 to increase sensitivity while decreasing specificity, which is more suitable for population-based disease screening. This added filter achieved a sensitivity of 91%, a specificity of 89%, and maintained an accuracy of 90%. The AI-based model showed greater discriminative power than metabolite-based and risk factor-based screening methods, as indicated by an AUC of 0.95 compared to 0.81 and 0.64 for the metabolite-based and risk-based methods, respectively. The model was also evaluated for its ability to distinguish between different pathological stages and exhibited higher accuracy in screening severe cases. The study reported improved interpretability of the mass spectra with this model. By ranking the top 10 images with positive importance scores and the top 10 with negative importance scores, the authors identified 9 metabolites out of the 20 image tiles. This study demonstrated that deep learning AI-based models can effectively distinguish between SN and SP cases for ESCC (Wang et al., 2023). Furthermore, the model improved interpretability by encoding multi-channel images to represent the entire metabolome, an important feat in the effort to prevent occurrences of “black boxes”. The findings indicate that similar deep learning methods could be applied to metabolomics-based clinical diagnosis (Wang et al., 2023). For real-world clinical applications, the model should be validated with a multi-center cohort. Additionally, since only MS1 data was used, incorporating MS2 data could further enhance the model.

### Salivary metabolomics for colorectal cancer detection (Kuwabara et al., 2022)

Kuwabara et al. aimed to identify metabolite biomarkers in saliva that can serve as diagnostic tools for the early detection of Colorectal Cancer (CRC). This study employed Capillary Electrophoresis-Mass Spectrometry (CE-MS) (Zhang & Ramautar, 2021) and LC–MS (Seger & Salzmann, 2020) to perform untargeted metabolomics on saliva samples. The samples were collected from patients diagnosed with CRC, adenoma (AD), and controls (HC), totaling 2,602 samples. These samples were composed of 235 CRC samples, 50 AD samples, and 2,317 HC samples.Approximately 400 µl of unstimulated saliva were collected from each participant. Untargeted metabolomic analysis was conducted using CE-MS and LC–MS. The LC–MS data was processed with Agilent MassHunter Qualitative Analysis (Shah et al., 2021), while CE-MS data was analyzed using MasterHands with integrated noise filtering. Subsequent analyses focused on polyamine LC–MS data. The dataset was randomly divided into training and validation sets, each comprising 1,301 subjects. Metabolites present in at least 95% of the samples and exhibiting a p-value greater than 0.05 between CRC and HC groups, based on a Mann–Whitney test (Rosner & Grove, 1999), were selected. Clustering analysis was performed using fold changes between HC and CRC. Partial Least Squares-Discriminant Analysis (PLS-DA) was utilized to assess the overall differences in metabolic profiles between HC and CRC groups. Two comparisons were made, (1) distinguishing individuals with either CRC or AD from HC, and (2) distinguishing those with AD or HC from those with CRC. For both scenarios, Alternative Decision Tree (ADTree) (Podgorelec et al., 2002) and Multiple Logistic Regression (MLR) (Sperandei, 2014) models were tested, referred to as ADTree1 and ADTree2, and MLR1 and MLR2, respectively.

A total of 23 metabolites were selected for clustering analysis based on fold changes. These included *acetylated polyamines*, *pyruvate*, *lactate*, *succinate*, *malate*, and four amino acids. PLS-DA identified *N-acetylputrescine* and *N-acetylspermine* as key discriminators between CRC and HC groups. Additionally, pathways related to amino/nucleotide sugar metabolism, alanine/aspartate/glutamate metabolism, and arginine/proline metabolism were found to be enriched. The two ADTree models underwent cross-validation (CV) using the training data. ADTree1 comprised 16 trees and 7 nodes, while ADTree2 included 12 trees and 8 nodes. ADTree1 achieved a discriminability of 0.933 for all data and 0.86 for CV, generalizing well with a validation set performance of 0.87. ADTree2 demonstrated a discriminability of 0.951 for all data and 0.879 for CV, also generalizing effectively with a validation set performance of 0.87. MLR1 and MLR2 models, based on three metabolites each, showed AUC values with p-values < 0.0001 (Kuwabara et al., 2022). The AUC values for the ADTree models were higher compared to the MLR models. ADTree1 and MLR1 both exhibited 68% sensitivity, while ADTree2 and MLR2 showed 60% sensitivity. However, all correlations from the MLR models lacked statistical significance. The study also demonstrated that the models' accuracy was not influenced by tumor location by comparing left- and right-sided CRC and not seeing significant variance between these sides. Additionally, MiR-21 was shown to have the ability to discriminate CRC from HC using saliva samples.

The study successfully identified novel metabolic biomarkers and demonstrated that ADTree algorithms can effectively be used to diagnose patients with CRC using saliva samples. ADTree was shown to have better performance than models like MLR, showing greater promise in clinical uptake for CRC patients (Kuwabara et al., 2022). The authors noted that the sample proportions do not reflect the actual prevalence of the diseases studied. There is also an age bias between the HC group and the other groups. Furthermore, the study did not explore the applicability of the findings to other cancer types (Kuwabara et al., 2022). Lastly, the sensitivity and specificity of CRC detection models still require improvement for future studies.

## Diabetes

### ML for identifying kidney dysfunction associated metabolites in type 2 diabetes patients (An et al., 2024)

Present methods for detecting kidney issues in Type 2 Diabetes (T2D) patients such as serum creatinine-based estimated glomerular filtration rate (eGFR) may be inaccurate due to confounding influences from factors irrelevant to kidney function, but ML approaches for identifying metabolomic biomarkers present a promising alternative. This study performed by An et al. aimed to explore different ML methods for distinguishing metabolomic profiles associated with reduced GFR, which leads to decreased and abnormal kidney function in diabetes patients (Smushkin & Vella, 2010). 1626 T2D patients from Liaoning Medical University First Affiliated Hospital served as the development cohort and 716 patients from Second Affiliated Hospital of Dalian Medical University served as the external validation cohort. The patients’ demographic and anthropometric information such as gender, age, smoking, drinking, weight, height, systolic blood pressure, and diastolic blood pressure were measured. In addition, clinical data like plasma creatinine (SCR), cholesterol, triglycerides, high-density lipoprotein cholesterol (HDL-C), and low-density cholesterol (LDL-C) were collected (Duncan et al., 2001; Jukema et al., 2005). *Acylcarnitines* in plasma and amino acids such as *citrulline* and *orthinine* were measured using LC–MS (Seger & Salzmann, 2020).

Subjects were divided into three categories of glomerular filtration rates (GFR): elevated, mild reduction, and severe reduction, where elevated reduction is associated with higher risk of kidney dysfunction (Kang et al., 2023). The contribution of each metabolite feature to group separation was evaluated using orthogonal partial least squares discriminant analysis (OPLS-DA). Classification of metabolites according to their associated GFR levels was done using four ML techniques: logistic regression (LR) (Sperandei, 2014), SVM (Noble, 2006), RF (Rigatti, 2017), and XGBoost (Moore & Bell, 2022). Feature selection, or elimination of redundant information, was done using zero-mean normalization and lasso regression. Shapley Additive exPlanation (SHAP) (Rodríguez-Pérez & Bajorath, 2020) values were utilized to analyze the ML results and understand the average marginal contribution of each feature to each prediction, calculated by considering all possible orders in which features could be added to the model. AUROC was used to measure the performance of the models (Çorbacıoğlu & Aksel, 2023). Higher AUC values (within 0.5–1) indicated a better ability to distinguish between samples (Çorbacıoğlu & Aksel, 2023). The area under the precision-recall curve was used to assess trade-offs between precision and sensitivity of the models when there were imbalances between positive and negative classes (Keilwagen et al., 2014). Higher values (within 0–1) indicated a higher proportion of correctly identified positive predictions and a minimal number of false positives (Keilwagen et al., 2014).

The XGBoost model demonstrated the best performance out of the four techniques. When nine *acylcarnitines* and *citrulline* were incorporated into the XGBoost machine learning model, AUROC increased significantly from 0.794 to 0.894 and the SHAP value of each feature was greater than zero. Higher levels of *citrulline* and short chain *acylcarnitines* are thus associated with lower GFR levels in T2D patients, and thus lower kidney functioning, indicating a higher risk of kidney problems. The frequent co-occurrence of T2D and kidney disease has prompted the need for tracking kidney function using *creatinine*-based glomerular filtration rates in T2D patients. However, *creatinine*-based GFR doesn't reflect much information during the initial stages of kidney disease, and metabolomic biomarkers may be a more reliable method of prediction. The effectiveness of this study’s prediction model could bring attention to the importance of primary and secondary prevention and inspire doctors to evaluate kidney function in high-risk patient groups (An et al., 2024). While the study may have profound impacts in the field of precision medicine, there are limitations that should be taken into consideration. First, *glycosylated hemoglobin* and *proteinuria* were not included in the results and analysis due to missing values. However, testing a version of the model with adjusted data for proteinuria in a Japanese cohort study did not significantly impact the association between biomarkers and eGFR levels, potentially indicating that model comprehensiveness may be minimally impacted by such variable selection. Second, the T2D patients were all inpatients, so the study cannot be generalized to non-hospitalized T2D patients. And third, the causal relationship between the variables cannot be proven due to the small-scale and cross-sectional nature of the data. Future studies could develop models on a larger dataset to improve generalizability and accuracy of findings.

### Integrated bioinformatics and tree-based ML techniques for metabolic biomarker discovery (Yagin et al., 2024)

Many existing ML methods used for the purposes of T2D lack accuracy in identifying metabolite features associated with disease progression, indicating a need for better models. This study proposed using tree-based ML algorithms integrated with bioinformatics tools to develop a stronger diagnostic model that identifies metabolomic biomarkers associated with T2D. These techniques are commonly used in analyzing omics data due to their effectiveness with high-dimensional data. The metabolomic data used in this study was obtained from the publicly accessible NIH Common Fund National Metabolomics Data Repository (NMDR). Additional data was acquired by screening 34 healthy individuals and 31 T2D patients, diagnosed as per the American Diabetes Association Standards of Medical Care Guidelines. The plasma samples were analyzed using mass-spectrometry-based metabolomics methods. Obtaining glucose, lipid, and metabolomic data involved collecting blood samples into fluoride, heparin, and EDTA tubes. Plasma, stored and isolated at –80° C, was prepared under LC–MS (Seger & Salzmann, 2020) via resuspension in a chilled methanol–acetonitrile-water solution and then was centrifuged. Surface-lying liquid was subjected to Ultra High-Performance Liquid Chromatography-Mass Spectrometry (UHPLC-MS) for the purpose of metabolite extraction and identification (Courlet et al., 2020). Multivariate and univariate statistical approaches like fold change, Partial Least-Squares Discriminant Analysis (PLS-DA) (Gromski et al., 2015) and false discovery rates (FDR) (Green & Diggle, 2007) were applied to the data to understand how metabolite biomarkers are associated with T2D. For multivariate analysis, the data was standardized using median normalization, the Pareto scale, and log transformation. Differences in metabolite levels between the two subject groups were evaluated using an independent t-test with p < 0.05. FDRs were calculated using the Benjamini–Hochberg method (Green & Diggle, 2007) to minimize the proportion of false positives among the significant results. The metabolomic data was also fed into three tree-based ML algorithms: XGBoost (Moore & Bell, 2022), Light gradient Boosting Machine (LightGBM) (Zhang et al., 2019) and Adaptive Boosting (AdaBoost) (Hao & Huang, 2023). Predictive performance was evaluated using metrics including accuracy, F1-score, positive predictive value (PPV), negative predictive value (NPV), sensitivity, and AUC (Çorbacıoğlu & Aksel, 2023; Erickson & Kitamura, 2021). ROC analysis describes the performance of the classifier in sensitivity and specificity. Metabolites with AUC values above 70% in univariate ROC were identified as important features while examining the predictive performance of each biomarker (Çorbacıoğlu & Aksel, 2023).

Univariate ROC Analysis revealed that 18 metabolites with AUC values > 0.70 were identified as possible diagnostic biomarkers for T2D, including *pyruvate*, *D-Rhamnose*, *AMP*, *pipecolate*, *Tetradecenoic acid*, *Dodecanediothioic acid*, *Prostaglandin E3/D3* (isobars), *ADP*, and *hexadecenoic acid.* PLS-DA VIP results revealed that *D-Rhamnose*, *GMP*, and *IMP* had the highest scores (VIP > 1.0) (Yagin et al., 2024). Applying multivariate classification models on the metabolomic data revealed further information. The XGBoost model’s performance was as follows: accuracy = 0.831, F1 = 0.845, sensitivity = 0.882, specificity = 0.774, PPV = 0.811, NPV = 0.857, and AUC = 0.887. The LightGBM model’s performance was as follows: accuracy = 0.800, F1 = 0.817, sensitivity = 0.853, specificity = 0.742, PPV = 0.784, NPV = 0.821, and AUC = 0.860. And lastly, the AdaBoost model’s performance was as follows: accuracy = 0.785, F1 = 0.806, sensitivity = 0.829, specificity = 0.733, PPV = 0.784, NPV = 0.786, and AUC = 0.844 (Yagin et al., 2024). The best performance among the multivariate classification methods was exhibited by the XGBoost model as indicated by its accuracy, specificity, PPV, and AUC values in distinguishing between the control and T2D patient groups (Yagin et al., 2024).

While other studies have successfully employed ML models to identify metabolomic profiles associated with T2D, there has been notable room for improvement in terms of predictive accuracy and our knowledge of which methods have better predictive power compared to others. The tree-based models utilized in this study incorporated regularization techniques to avoid overfitting and capturing non-linear relationships, which are suitable for complex biological processes like disease progression. The study also suggests that XGBoost, compared to other tree-based ML methods, may provide an advantage considering its ability to accurately discover metabolomic biomarkers (Yagin et al., 2024). This study also goes further than discovering biomarkers by evaluating the predictive performance of each biomarker. The results of this study could help inform doctors’ decisions when dealing with high-risk patients to help prevent or treat T2D (Yagin et al., 2024). The complex nature and interactions of metabolites identified in this study with other systems and dependance on factors potentially unrelated to T2D poses a challenge unexplored in this study. Additionally, the study only observed metabolomic profiles of patients within the database, but more information could have been used to accurately determine the relationship between metabolite levels and T2D such as age, gender, BMI, medications, and other omics data. Prospective studies could be done following similar approaches but also incorporating this additional data as model inputs for increased accuracy and broader clinical application.

### ML and longitudinal metabolomics for biomarker discovery in gestational diabetes mellitus (Lu et al., 2023)

Longitudinal studies have the potential to help us better understand changes in metabolite levels associated with gestational diabetes mellitus (GDM) progression, a type of diabetes that develops during pregnancy. This study aimed to use traditional statistical and ML methods to determine biomarkers correlated with GDM and, in turn, gain insights into how to prevent or treat GDM (Buchanan & Xiang, 2005). The longitudinal metabolomics study was conducted at a GDM care center in the Fifth People's Hospital of Shanghai. Subjects consisted of Chinese pregnant women. 30 of the sampled patients were diagnosed with GDM and 30 were healthy. Metabolomic data was recorded across each of the patients’ three trimesters of pregnancy. All women were similar in pre-pregnancy BMI and age (patients with GDM were 30.6 ± 3.57 years of age, and patients in the control group were 30.9 ± 3.57 years of age). Some patients were excluded from the study if they had pre-existing conditions such as any infectious diseases within two weeks of serum sample collection, dysfunctions in the liver or kidney, viral infections, malignant tumors, or any pre-existing pancreatic exocrine diseases. GDM diagnoses were based on 2020 ADA guidelines. A total of 180 blood serum samples were obtained from 60 participants at three different periods of pregnancy: at the first obstetrics clinic visit (14.1 ± 3.1 weeks), the second trimester (25.5 ± 1.9 weeks), and at the third trimester before delivery (36.0 ± 2.1 weeks). Data collected included blood cell count and biochemical parameters. Gestational age, blood pressure, and anthropometric parameters were recorded, and patients were asked to fill out a questionnaire about their last menstruation, diabetes/GDM family history, pre-pregnancy height/weight, method of conception, and parity. eGFR was determined using the CKD-EPI (Chronic Kidney Disease Epidemiology Collaboration) equation. The 180 serum samples, stored at –80° C. were thawed on ice and vortexed for ten seconds. 50 μL of sample and 300 μL of extraction solution were added into a 2 ml microcentrifuge tube and then vortexed for 3 min and centrifuged at 4° C for 10 min. Supernatants were then collected and placed at –20° C for 30 min and centrifuged at 4° C for three minutes. Lastly, 180 μL of the supernatant were collected for LC–MS/MS analysis (Seger & Salzmann, 2020). The LC–MS/MS data was converted into mzML format using Proteo-Wizard software. Differences in metabolic data between the two groups of patients across trimesters were analyzed using FC, or the ratio of peak area between the two groups and t-test with FDRs (Green & Diggle, 2007), where FDR < 0.05 was considered statistically significant (Boca & Leek, 2018). PCA was used to identify significant components explaining variance. The relative importance of each variable in each prediction was calculated using OPLS-DA. Trimester-specific metabolic characteristics were observed from metabolite intensities across patients with and without GDM across the three trimesters. The GDM and control patient data were compared within each trimester separately, ensuring no subject-specific data leakage across time points, and metabolite data were compared across trimesters to observe trends across time to observe correlations between metabolite changes and other clinical trends. Pearson correlation coefficients and linear regression was used to determine the correlation between blood glucose and metabolite levels. Support Vector Regression (SVR) (Noble, 2006) was used to correct peak area (all peaks with deletion rate greater than 50% were eliminated). Since traditional statistical analyses have the potential to produce false positive results, ML workflows such as bootstrap resampling with 1000 iterations and metabolite variable selection using LASSO were utilized to identify GDM biomarkers (Xi et al., 2023). Metabolites were annotated using the Human Metabolite Database (HMDB) (Wishart et al., 2022) and the Kyoto Encyclopedia of Genes and Genomes (KEGG) database (Kanehisa & Goto, 2000).

Significant differences between the control and GDM groups across the three trimesters were manifested by 32 metabolites. OPLS-DA analysis revealed major distinctions in the metabolic profiles of GDM and control groups across different trimesters, with R^2^ Y values exceeding 0.9, indicating that up to 90% of the variance in the data could be explained by the models. The results from permutation tests further confirmed that OPLS-DA models were not overfitting. For longitudinal analysis of metabolites across all three semesters, hierarchical clustering was applied to group the metabolites into observable patterns of change. The clustering heatmap of these metabolites revealed that cluster 1 metabolite levels decrease in the GDM group, cluster 2 metabolite levels fluctuated slightly across different trimesters in the GDM group and increased within the control group, and cluster 3 metabolite levels increased throughout the pregnancy. There was a significant correlation between *glycerophospholipids* and GDM, including *phosphatidic acid* (PA), *lysophosphatidic acid* (LPA), *phosphatidylcholine* (PC), *lysophosphatidylcholine* (LPC), *phosphatidylethanolamine* (PE), and *lysophosphatidylethanolamine* (LPE). Within the 1000 bootstrap samples, *allantoic acid* showed the highest stability in the first trimester, *3-Aminopiperidine-2,6-dione* showed the highest stability in the second trimester, and *lysine* had the highest stability in the third trimester. Overall, *allantoic acid* (a byproduct of *uric acid*) demonstrated the best association with GDM in early and mid-pregnancy, with AUC values of 0.989 and 0.899 in the first and second trimesters respectively. This longitudinal study provided insights into the metabolic signatures associated with various stages of GDM over the course of a pregnancy cycle (Lu et al., 2023). This data can be used to predict the onset of disease early on during pregnancy, helping prevent or treat it prior to the condition gets critical. This study also revealed the clinical uptake potential of LASSO use and bootstrap resampling to eliminate false positives and identify these metabolic biomarkers in their association to GDM (Lu et al., 2023). Due to the small sample size used, future studies may need to replicate on a larger dataset to generalize findings to large-scale populations. Additionally, many of the fold change values of different metabolites approached one, indicating the results’ limited biological significance despite statistical significance.

### Diagnosing diabetic retinopathy in T2D using XAI (Yagin et al., 2023)

Diabetic retinopathy (DR) is a microvascular complication which is the most significant factor underlying T2D-related vision loss (Yagin et al., 2023). This study’s main goal was to address the need for early DR diagnosis using the explainable artificial intelligence (XAI) framework on metabolomic data (Pandit et al., 2023; Zhang et al., 2022). Using only traditional statistical methods to identify biomarkers for DR has proven to be ineffective due to the disease’s complex development, resulting in confounding factors and model overfitting. Regardless of accuracy, many existing classification methods are difficult to interpret and therefore determine prediction reliability. XAI’s main benefit is its enhanced interpretability regardless of output accuracy, allowing the developer to identify specific areas for improvement. This newfound capability could help with refining an unreliable model into a more reliable one.Their primary benefit here is enhanced transparency and hypothesis generation rather than guaranteed improvements in predictive performance. Clinical, biochemical, and metabolomic biomarkers classified under one of the following categories were studied: non-DR (NDR), non-proliferative diabetic retinopathy (NPDR), and proliferative diabetic retinopathy (PDR) (Pandit et al., 2023). The dataset consisted of 317 T2D patients (143 NDR patients, 123 NPDR patients, 51 PDR patients) with 145 features (metabolites and other factors important for determining disease), where 10% of the data was used for validation and 90% was used for discovery. All the patients’ gender, age, height, weight, BMI, glucose, and creatinine levels were recorded.

Blood serum samples were collected from the T2D patients with/without DR and stored at –80° C. 122 metabolites were associated with DR and used for additional statistical studies. The validation dataset was used for hyperparameter optimization and feature selection whereas the discovery dataset was used for model training and performance evaluation. Hyperparameter optimization was done using Bayesian optimization techniques and model performance was measured using a ten-fold cross validation technique, in which the discovery dataset was split into 10 subsets or folds, and the model was trained 10 times using a different fold as the test set each time. The remaining 10% of data reserved for validation was used to check against overfitting during the feature selection and hyperparameter tuning phase. Feature selection and classification algorithms were combined to determine the significance of biomarkers tied to different DR subclasses in T2D patients. The minimum redundancy and maximum relevance (mRMR) (Alshamlan et al., 2015) technique and Boruta method were used for feature selection, and explainable boosting machine (EBM) (Körner et al., 2024) was utilized for feature extraction. mRMR selects features relevant to class labels by removing features that have minimal correlation with one another. Boruta uses an RF classifier to calculate z-scores, which is necessary for iteratively eliminating irrelevant features and determining the most important ones. The final framework incorporated ML models XGBoost (Moore & Bell, 2022), natural gradient boosting (NGBoost) (Hussain et al., 2021), and EBM (Körner et al., 2024) for classification. The models were evaluated on accuracy, precision, recall, F1-Score, and AUROC (Boca & Leek, 2018). Since the classification task was multi-class, these evaluation metrics were macro-averaged across NDR, NPDR, and PDR. AUROC was computed on a one-vs-rest strategy for each class and then averaged.

The best performance was observed when XGBoost was used for classification and EBM was used for feature selection (accuracy = 82.16 ± 1.71, precision = 82.47 ± 1.61, recall = 82.16 ± 1.61, F1 = 82.32 ± 1.86, AUROC = 89 ± 0.17) (Yagin et al., 2023). With this combination of models, six significant biomarkers were found in relation to diagnosis of DR: tryptophan *(Trp)*, *phosphatidylcholine diacyl C42:2* (PC.aa.C42.2*)*, *butyrylcarnitine* (C4), *tyrosine* (Tyr), *hexadecanoyl carnitine* (C16) and total *dimethylarginine* (DMA) (Yagin et al., 2023). Studying these resulting biomarkers may provide us with additional insights into the progression of DR, inspiring the development of more personalized and cost-efficient diagnostic tools (Yagin et al., 2023). Thus far, studies correlating blood metabolites with DR are limited. Since several factors affecting the progression of DR make it difficult to distinguish important biomarkers using statistical approaches, taking a hybrid approach that utilizes the predictive power of XGBoost and the explainability of EBM may offer a better alternative to processing high-dimensional metabolomic data to evaluate patient health. Ultimately, this study proposes a novel and potentially powerful method of diagnosing DR early on to minimize the progression of the disease and identify individualized treatment strategies (Yagin et al., 2023). The efficacy of the model on datasets other than the one it was trained on is unknown, so there is the potential for overfitting. External validity of the ML model should be confirmed via testing on additional datasets. Prospective studies could also incorporate multi-omics data besides metabolomic data to more accurately classify DR and explore interactions between metabolomic and other omics data.

### Metabolomic and proteomic biomarkers for diabetic kidney disease (Liu et al., 2021a)

Diabetic kidney disease (DKD) (Bonner et al., 2020) is a microvascular complication commonly associated with T2D mellitus (2-DM) (Leiherer et al., 2024). The current most frequently used resources for diagnosing DKD are urine and kidney biopsy samples. However, there may be up to a 49.2% chance of inaccurate DKD diagnosis if using clinical information alone. This study proposes the usage of proteomic and metabolomic data in blood to predict the onset and examine the progression of DKD, which could lead to earlier diagnosis and more accurate detection of DKD compared to past traditional methods. 1,513 participants, including both healthy adults and 2-DM, early-stage DKD (DKD-E) and advanced-stage DKD (DKD-A) patients, were recruited from four different medical centers to take part in the study. All recruited 2-DM patients were clinically diagnosed for at least ten years. The participants were divided into one discovery and four testing groups. Patients from the primary center in NJ were designated the discovery cohort and included 1,102 individuals. 174 candidates within the primary center were set aside as the testing cohort. 237 participants were recruited from the other three subcenters and designated as three external testing cohorts. Demographic and clinical characteristics of the different participant cohorts were collected. The discovery cohort included 513 females and 589 males from 20–75 years old with BMI 16.22–40.7 kg/m^2^. *Hemoglobin A1c*, blood glucose, *cholesterol triglyceride*, *high-density-lipoprotein*, and *low-density lipoprotein* levels were similar among discovery cohort patients, but DKD-A patients had decreased eGFR, elevated blood urea nitrogen levels, *serum creatinine*, and *urinary albumin* when compared with 2-DM and DKD-E patients (Liu et al., 2021a). In the discovery cohort, 30 serum samples were randomly selected from each group and protein biomarkers were identified using proteomics. Multiple Affinity Removal Column Human 14 was used to remove the 14 most abundant proteins from the serum samples, around 200 μg of these proteins were processed following filter-aided sample preparation (FASP), and UV light spectral density of 280 nm was used to estimate peptide content. Tandem mass tag (TMT) was used to label around 100 μg of peptide mixture reagent, and then the TMT-labeled digest samples were divided into twelve fractions by an increasing acetonitrile step-gradient elution. For 60 min, LC–MS/MS (Seger & Salzmann, 2020) analysis was performed on a Q Exactive mass spectrometer coupled with an Easy nLC. A data dependent top10 method was used to obtain MS data. Resolution and other factors involved in acquiring the data were optimized to ensure accuracy and sensitivity. The metabolite levels of the discovery cohort were also measured. Serum samples were mixed with methanol and cold MTBE, vortexed, and processed at 4° C. Following centrifugation, the supernatant was dried, derivatized with *methaxyamine hydrochloride*, and treated with BSTFA. GC–MS (Garcia & Barbas, 2011) analysis was then performed, and the raw data was converted into ABF format for further processing. The data was finally normalized using MetaboAnalyst 3.0 and significant metabolites were identified using multivariate and univariate analysis methods. Biomarker results were validated using the testing cohorts. The proteome and metabolome, determined using differential expression analyses on the sera of the discovery cohort, were used to train five different ML models. A non-parametric Wilcoxon rank-sum test was performed on each metabolite in the metabolomic data to identify differentially expressed metabolites by pairwise comparisons. A five-fold cross validation was performed on all pairwise predictions and ML algorithms such as LDA (Hu et al., 2020), SVM (Noble, 2006), RF (Rigatti, 2017), LR (Sperandei, 2014), and PLS-DA (Gromski et al., 2015) were applied to the testing cohorts for predicting DKD progression. Lastly, the proteomic and metabolomic data was combined to perform integrative analyses and determine proteins or metabolites that serve as biomarkers for diagnosing DKD (Liu et al., 2021a).

The proteomic data revealed that *α2-macroglobulin*, *cathepsin D*, and *CD324* could be used as proteomic biomarkers in monitoring the progression of DKD (Liu et al., 2021a). The metabolomic analysis, which identified 349 serum metabolites, identified *galactose* metabolism and *glycerolipid* metabolism as having significant associations with disturbance in DKD, and *glycerol-3-galactoside* may be a biomarker for predicting the onset of DKD. All five of the ML models demonstrated similar results, so RF was chosen to display the results. Although urine and kidney biopsy samples are usually used to diagnose DKD, oftentimes they do not reveal much information until DKD has progressed to a certain degree. Thus, the study concluded that proteomic and metabolomic data can increase our ability to predict and monitor DKD progression (Liu et al., 2021a). A high diagnostic value measured via a blood sample could serve as evidence of DKD onset, allowing for preventative and treatment measures to be taken early on before severe disease manifestation in patients. The study’s considerable contributions to DKD diagnosis should be considered with future improvements to study design and unexplored factors. First, kidney damage may be present in T2D patients with normal microalbuminuria, but the microalbuminuria levels in most of the dataset’s DKD-E patients were normal, so important proteome or metabolome-based values may have been missed. Second, further ML analysis may be necessary to more accurately identify multi-omics biomarkers. Third, certain types of lipids and amino acids were excluded from analysis in the blood serum samples, and including these molecules can yield more accurate results. Fourth, the proteome and/or metabolome of non-diabetic CKD patients were not examined, so changes in metabolite/protein levels may be due to diabetes itself rather than absolute nephron loss (Liu et al., 2021a). Prospective studies should compare urine and serum samples to understand the effect of confounding variables.

### ML for discovering T2D metabolomic biomarkers (Leiherer et al., 2024)

The co-occurrence of T2D and cardiovascular disease (CVD) is well-studied, as CVD is the leading cause of death in T2D patients (Leiherer et al., 2024). However, there is still much to be learned about applications of ML to metabolomics data for disease prevention purposes. This study’s objective was to apply ML approaches to targeted metabolomic data in cardiovascular risk patients to predict the 4-year risk of developing T2D. 279 Caucasian subjects initially free of T2D but at risk for cardiovascular diseases underwent coronary angiography and were utilized for gathering anthropometric and targeted metabolomic data over the course of four years. Venous blood samples were taken from patients after twelve hours fast and tested following traditional laboratory procedures. Serum samples were taken and frozen at –80° C to allow for metabolomic analysis while protecting against repeated freeze–thaw cycles. 407 baseline patient samples were selected for targeted quantitative metabolomics analysis using LC–MS (Seger & Salzmann, 2020) and flow injection analysis (FIA-MS) (Yue et al., 2022). 535 total compounds were analyzed in total. 11% of the participants developed T2D during the four-year period, with diabetic status being examined at the recruitment phase and at a follow-up four years later. Besides glucose levels, there were no statistically significant differences between those who developed T2D and those who did not. Both groups were prediabetic as per their HbA1c levels. The metabolomics dataset consisted of over 500 metabolites (Leiherer et al., 2024). Statistical analysis methods such as chi-squared tests and Jonckheere-Terpstra tests for continuous variables were performed to identify differences in baseline characteristics. Recursive feature elimination (RFE) (Escanilla et al., 2018) via algorithms like TreeBag, Caret, RF, and Naive Bayes were used to identify feature importance. ML models were evaluated based on true positive, false positive, true negative, and false negative values, which were used to determine precision, accuracy, specificity, balanced accuracy, and F1 score (Erickson & Kitamura, 2021). SHAP (Rodríguez-Pérez & Bajorath, 2020) was also used to determine feature contribution to the final prediction.

The most important features as identified by the ML algorithms were *hexoses*, amino acids (*glycine*, *isoleucine*, amino acid derivative *kynurenine*), and bile acids *(chenodeoxycholic acid (CDCA)* and *deoxycholic acid, DCA*) (Leiherer et al., 2024*).* SVM with linear kernels yielded the highest performance (F1 score = 0.50, specificity = 93%, balanced accuracy = 0.72, unbalanced accuracy = 0.88) out of the ML models used (Leiherer et al., 2024). This study successfully employs ML to analyze metabolomic data, indicating its potential for identifying individuals at risk of developing T2D in further studies. This four-year study provides a longitudinal perspective on the association between metabolomic profiles and risk for T2D, which allows for greater knowledge on disease etiology and progression (Leiherer et al., 2024). Mapping out the metabolomic signatures along various points within a four-year period could help reveal patterns between disease and metabolite levels, which can be used for early detection of T2D and in turn prevention of long-term complications such as cardiovascular disease, neuropathy, and more.

Since patients were of the same race (Caucasian) and were all at increased risk for cardiovascular diseases, the results of this study may not be applicable to patients of other demographics. Additionally, the model that was trained was only validated by a single test set generated from the whole dataset; validation on a separate cohort with similar metabolomic profiles should be done for further model improvement. Certain factors for determining the risk of developing diabetes such as HbA1c were also not accounted for when predicting T2D incidence throughout the four-year period, although these factors are tied to the glucose levels accounted for by metabolomic analysis. Lastly, it is uncertain whether the patients perfectly adhered to their prescribed medical treatment instructions. To yield more reliable results, future studies could be done following similar approaches while also addressing these limitations.

## Other conditions

### Biomarkers in neovascular age-related macular degeneration via iRF (Künzel et al., 2024)

This research paper explored the use of iterative random forests (iRF) to analyze peripheral blood xenobiotic profiles and discover potential biomarkers associated with clinical phenotypes of neovascular age-related macular degeneration (nAMD) (Künzel et al., 2024; Shao et al., 2016). Age-related macular degeneration causes irreversible visual impairment and is linked with major risk factors including advancing age, genetic predispositions, and environmental exposures (Shao et al., 2016). The focus of this study was to further understand the environmental contributions to nAMD through the analysis of xenobiotics, which are external compounds the body is exposed to Künzel et al. (2024). While genetic and age-related factors in nAMD have been studied extensively, the role of xenobiotics remains underexplored in current literature. By investigating these external compounds, the researchers aimed to shed light on how environmental exposures may influence the pathogenesis of nAMD and contribute to its clinical variability.

The authors employed a cross-sectional observational design to investigate the xenobiotic profiles of patients with nAMD. Their study included 46 patients diagnosed with nAMD, undergoing anti-vascular endothelial growth factor (anti-VEGF) intravitreal therapy (IVT). These patients were stratified into two groups based on choroidal neovascularization (CNV) activity levels: 25 patients with chronically active CNV (CAC) and 21 patients with effectively controlled CNV (ECC) (Fleckenstein et al., 2021; Shao et al., 2016). Importantly, the study did not include a healthy control group (non-nAMD), which limits the ability to determine whether the identified xenobiotic profiles are unique to nAMD or reflect inter-individual variation in environmental exposures. Clinical examinations, including optical coherence tomography (OCT), fundus autofluorescene (FAF), and fluorescein angiography (FA), were performed alongside extensive history-taking to collect demographic and clinical data. Data points from clinical examinations and blood samples were collected for further xenobiotic metabolite analysis, and 156 xenobiotic metabolites were quantified using LC–MS (Thomas et al., 2022).

The 156 xenobiotic features identified from LC–MS were then modeled with iRF, which is a multivariate approach that iteratively refines feature selection (Basu et al., 2018). Individual metabolites served as predictors and clinical phenotypes as response variables. The iRF model-built classification trees and ranked the features based on their importance in predicting the associated clinical phenotype using the Gini coefficient for each clinical phenotype e.g., CNV activity, subretinal fluid (SRF), subretinal hyperreflective material (SHRM), and intraretinal cysts (IRC) (Künzel et al., 2024). This iterative process was designed to progressively eliminate irrelevant features and focus on the most predictive xenobiotics. The models were trained and tested on a 50:50 data split, with 500 trees per random forest and up to twenty iterations per model. However, the small effective test set size (n ≈ 23 per group) introduces high variance in performance estimates and raises the risk of overfitting. Large external validation cohorts will be necessary to confirm generalizability. Key metrics such as the AUC score (Çorbacıoğlu & Aksel, 2023), were used to evaluate model performance across iterations (Künzel et al., 2024). Additionally, the xenobiotics were classified into six categories (e.g., food, drug metabolism, benzoate metabolism) and their associations with specific clinical outcomes, such as SRF presence, were analyzed using statistical methods, including the Mann–Whitney U-test (Rosner & Grove, 1999) and the Benjamini-Hochberg (Green & Diggle, 2007) procedure for false discovery rate correction. The iRF models effectively reduced the number of xenobiotic features by prioritizing the most predictive biomarkers across multiple iterations.

After twenty iterations, feature selection stabilized, with best performance for predicting CNV activity. Among the 156 features, *Perfluorooctanesulfonate (PFOS*) and *Ethyl β-glucopyranoside* emerged as the most significant biomarkers across three major clinical phenotypes: CNV activity, IRC, and SHRM (Chang et al., 2016; Künzel et al., 2024). Additional metabolites, such as *Saccharin*, *Quinate*, and *Paraxanthine*, were identified as important for other specific phenotypes, including IRC and SHRM. Drug-related metabolites showed a significant association with the presence of subretinal fluid (SRF), even though the number of different drug-related metabolites in patients' blood was low. This finding suggests a potential link between certain drug metabolites and SRF presence in nAMD patients (Künzel et al., 2024). While accuracy was high for CNV activity, performance gains for SRF were limited, which is likely due to the small sample size. The results highlight the capability of iRF models to efficiently select relevant xenobiotics from complex data and identify potential biomarkers for nAMD. The identification of PFOS and other metabolites opens new avenues for understanding the role of environmental xenobiotics in the progression of nAMD and could inform future strategies for personalized treatment (Künzel et al., 2024). Future studies may need to increase sample size and include external validation of xenobiotic markers to fully be able to generalize findings.

### COVID-19 metabolic biomarkers identified by ML (Elgedawy et al., 2024)

This study aimed to identify potential biomarkers for COVID-19 (Sharma et al., 2021) severity and outcomes by analyzing stage-specific metabolite changes using targeted metabolomics. ML and statistical models, including RF (Rigatti, 2017), PLS-DA (Gromski et al., 2015), OPLS-DA (Boccard & Rutledge, 2013), and SVM (Noble, 2006), were employed to classify patients based on disease severity and predict outcomes, ultimately enhancing diagnostic and prognostic accuracy compared to current methods in literature. The study used a cross-sectional observational design to explore metabolic changes in 295 participants, including 99 healthy controls, 100 non-severe, 46 severe, and 50 critical COVID-19 patients. All patients were confirmed to be COVID-19 positive via PCR tests and stratified by disease severity. Blood samples from all subjects underwent UPLC-MS (Plumb et al., 2006) to profile 50 targeted metabolites, with normalization and log transformation. The metabolomic data was normalized and log-transformed for further analysis. RF was used to classify subjects based on metabolite concentrations and disease severity, with models built using an ensemble of 1000 trees. The performance of the RF model was evaluated using accuracy, sensitivity, and specificity scores. PLS-DA and OPLS-DA aided in visualizing the separation between patient groups and identifying important metabolites that contributed to group differentiation. The OPLS-DA model was validated with parameters such as R2Y (explained variance) and Q2Y (predictive ability). SVM was employed to explore various combinations of features, including key metabolites and blood parameters, to improve prediction accuracy for critical COVID-19 cases. Univariate statistical tests, such as Student’s t-tests and ANOVA (Kim, 2017), were performed to assess the significance of metabolite concentration differences between groups. Additionally, pathway enrichment analysis was conducted using MetaboAnalyst 5.0 (Pang et al., 2021) to map the differentially expressed metabolites to known metabolic pathways. Blood parameters (such as ferritin, IL-6, D-dimer) were also incorporated into the analysis to enhance the predictive power of the models by providing additional clinically relevant data points that are known to correlate with COVID-19 severity and outcomes. These parameters complement metabolomic data by offering a broader representation of the biological processes and immune responses at play during infection, thereby improving the models’ ability to distinguish between different clinical groups. PCA (Groth et al., 2013) was applied to detect and remove outliers and cross-validation techniques were used to optimize the ML models.

The ML models identified distinct metabolite signatures corresponding to different stages of COVID-19 infection. Among the tested approaches, the RF model demonstrated superior performance, achieving an accuracy of 97% in distinguishing between healthy individuals and COVID-19 patients (Elgedawy et al., 2024). Key metabolites such as *arginine*, *malonyl methylmalonyl succinylcarnitine*, and *tauroursodeoxycholic acid* were found to be significant biomarkers for disease onset, progression, and critical illness. Pathway enrichment analysis highlighted bile acid biosynthesis and amino acid metabolism as critical pathways in their association to disease progression. The researchers also demonstrated that combining metabolomics data with blood parameters improved prediction accuracy, achieving accuracy up to 91% with an AUC of 0.9 in specific scenarios. This study provides valuable insights into the metabolic alterations that occur during COVID-19 infection and identifies key metabolites that could serve as potential biomarkers for diagnosis and prognosis. The integration of metabolomics with ML offers a robust tool for patient stratification, which could contribute to the development of personalized treatment strategies, thereby improving clinical outcomes in COVID-19 management (Elgedawy et al., 2024). However, the lack of external validation for the identified biomarkers limits the generalizability of findings. Additionally, the small cohort size, particularly for severe and critical patient groups, might reduce the robustness of the results. Future studies with larger, independent cohorts are necessary to validate these findings and expand their clinical applicability.

### Metabolomics assay for embryo implantation prediction (Cabello-Pinedo et al., 2024)

The objective of this study was to develop and validate a non-invasive metabolomics assay, called the Metabolite Pregnancy Index (MPI), to predict the implantation potential of embryos in in-vitro fertilization (IVF) (Choe & Shanks, 2024). The research sought to identify specific metabolite biomarkers in embryo culture media that correlate with successful implantation, thereby providing a predictive model that could improve embryo selection accuracy. The study reports a dataset of 218 participants overall, with 101 embryo culture media samples analyzed in the discovery phase and 169 in the validation phase. While these figures added to more than 218, the authors did not explicitly clarify this discrepancy; it may reflect multiple embryos derived from the same participants across the two phases (Cabello-Pinedo et al., 2024). Each sample corresponded to a blastocyst-stage embryo with a known implantation outcome. The study utilized a case–control design with two phases: an initial discovery phase and a subsequent validation phase. In the discovery phase, spent embryo culture media from 101 embryos with known implantation outcomes were collected. An untargeted metabolomics approach was employed using Ultra-High Performance Liquid Chromatography (Cielecka-Piontek et al., 2013) coupled with an Orbitrap Fusion Tribrid Mass Spectrometer (Espadas et al., 2017) (UHPLC-OT-FTMS) to detect and quantify over 7,500 small molecules in the media. Statistical analyses, including volcano plots and fold change measurements, were applied to filter these metabolites down to 148 with significant correlations to implantation outcomes. Each metabolite was further evaluated for structural information and its contribution to implantation prediction, resulting in the selection of 47 candidate biomarkers associated with either successful or failed implantation outcomes. To characterize the biological significance, pathway enrichment analyses using Mummichog (Zhang & Horvath, 2005) and Gene Set Enrichment Analysis (GSEA) (Subramanian et al., 2005) were performed, revealing enrichment in several pathways (Cabello-Pinedo et al., 2024).

For the validation phase, a targeted metabolomics approach was applied using the Triple Quadrupole Mass Spectrometer (UHPLC-TSQ) (Espadas et al., 2017). An optimized multiple-reaction monitoring (MRM) method, which allows selective detection of predefined metabolite fragments with high sensitivity and reproducibility, was developed to improve accuracy of the biomarkers identified in the discovery phase. Of the 47 biomarkers carried forward, only 36 could be consistently detected and quantified with sufficient accuracy and reproducibility, and these were retained for the final MPI model. The MPI, derived from the concentration of the 36 validated biomarkers, is an index score ranging from 0 to 1, with higher values indicating a greater likelihood of embryo implantation. The MPI model was developed using the Fusion Method, an AI-based technique that integrates results from multiple predictive algorithms (Huang et al., 2023). To ensure robustness and prevent overfitting, the model underwent k-fold cross-validation (Jung & Hu, 2015). Accuracy, PPV, and NPV were calculated to assess the model’s performance (Erickson & Kitamura, 2021). The predictive model achieved 85% accuracy, with a PPV of 88% and a NPV of 77% on the test samples, indicating strong predictive capability for implantation outcomes. Key metabolic pathways, including *tryptophan*, *arginine* and *proline* metabolism, and *lysine* degradation, were significantly enriched in the identified biomarkers, suggesting a metabolic basis for embryo implantation potential. Notably, the MPI score showed a strong correlation with clinical implantation outcomes, reinforcing its utility as a robust tool for non-invasive embryo selection (Cabello-Pinedo et al., 2024). This study introduced a non-invasive, metabolomics-based assay that improves embryo implantation predictions, providing a tool that could enhance the success rates of IVF by prioritizing embryos with high implantation potential without requiring invasive procedures like embryo biopsy. The high PPV suggests that this assay can reliably identify embryos with better chances of implantation, potentially reducing the need for multiple embryo transfers and increasing successful pregnancies in IVF treatments (Cabello-Pinedo et al., 2024). The study’s single-center design and lack of external validation across varying IVF centers leaves valuable room for improvement in future studies to optimize generalizability. Additionally, the sample size was limited for non-implanting embryos, which may affect the robustness of findings for non-pregnancy outcomes. Future studies with larger, multi-center cohorts are needed to validate these findings and assess applicability to various embryo culture conditions and media types.

### Biomarkers for tuberculosis and nontuberculous mycobacteria via metabolomics and ML (Anh et al., 2024)

This study aimed to identify urinary metabolic biomarkers that can distinguish between tuberculosis (TB) and nontuberculous mycobacteria (NTM) infections (Bloom et al., 2017; Koh, 2017). By analyzing the differences in urinary metabolomic profiles, the researchers sought to develop a non-invasive diagnostic tool using ML to classify these two pulmonary diseases. This approach shows potential to improve diagnostic accuracy in differentiating TB from NTM, as both conditions are difficult to distinguish clinically due to overlapping respiratory symptoms. Conventional methods such as sputum culture, while considered the gold standard, are time-consuming, may have limited sensitivity in paucibacillary disease, and can contribute to diagnostic delays or misclassification (Anh et al., 2024).

This study used urine samples from 54 patients, comprising 35 TB patients and 19 NTM patients. These samples were collected before antimicrobial treatment, allowing for a baseline metabolic profile of each group. Metabolomic profiling was conducted through an untargeted approach using UPLC-MS system coupled with a Quadrupole time-of-flight mass spectrometer (Chernushevich et al., 2001) Data preprocessing and alignment were carried out using MS-DIAL, followed by statistical analysis in MetaboAnalystR. The study employed univariate and multivariate methods, such as PCA (Groth et al., 2013) and PLS-DA (Gromski et al., 2015), to explore the variance and differentiate between TB and NTM. For biomarker identification, five ML models were applied: RF (Rigatti, 2017), SVM (Noble, 2006), XGBoost (Moore & Bell, 2022), Neural Network (NN) (Zou et al., 2008), and k-NN (Ehsani & Drabløs, 2020). The performance of each model was evaluated using a nested five-fold cross-validation, focusing on metrics such as the AUC of the ROC curve (Çorbacıoğlu & Aksel, 2023).

The study identified 6 key urinary metabolites: *methionine*, *valine*, *glutarate, 3-hydroxyanthranilate*, *corticosterone*, and *indole-3-carboxyaldehyde*. These metabolites identified through univariate ROC analysis (AUC ≥ 0.7, p-value < 0.05) and validated using a voting strategy across ML models, emerged as robust molecular signatures for distinguishing NTM and TB. Traditional linear models, such as PCA and PLS-DA, demonstrated limited performance in capturing the metabolic differences between NTM and TB groups, as reflected in their low explained variance (e.g., less than 20% in PCA) and poor predictive metrics (e.g., PLS-DA accuracy = 0.72, Q^2^ = 0.17) (Anh et al., 2024). In contrast, ML models, particularly the RF model, excelled with an AUROC of 0.828 ± 0.101, showcasing their ability to manage high-dimensional, complex data effectively. This performance gap highlights the added value of ML models in biomarker discovery for this dataset.

These findings suggest that metabolomics, when integrated with ML algorithms, may offer a promising non-invasive adjunct to traditional diagnostic tools, especially in cases where standard microbiological tests are time-consuming or inconclusive. However, the authors appropriately caution that the small sample size (n = 54) and lack of external validation limit the generalizability of their results. Moreover, the AUROC values observed are still below those reported for optimized molecular assays, such as the Xpert MTB/RIF assay, which has shown AUCs ranging from around .947 to 0.969 in differentiating pulmonary TB cases (Liu et al., 2021b). As such, metabolomics is unlikely to fully replace sputum-based molecular diagnostics but may serve as a useful complementary tool, particularly in scenarios where rapid, non-invasive screening is desirable. Further validation in larger, multicenter cohorts will be essential to confirm these results and evaluate their clinical applicability.

### ML and lipidomics reveal pre-metabolic and metabolic syndrome biomarkers (Huang et al., 2024)

This study’s objective was to identify plasma lipid biomarkers that can distinguish between pre-metabolic syndrome (pre-MetS) and metabolic syndrome (MetS) using a nontargeted lipidomics approach (Eckel et al., 2005; Huang et al., 2024). These conditions comprise a set of cardiometabolic risk factors such as high blood sugar and hypertension, among others, with pre-MetS comprising metabolic irregularities but not meeting the full criteria to be diagnosed as Mets (Huang et al., 2024). The researchers sought to construct predictive models that could improve early detection and characterization of these metabolic conditions through ML techniques, ultimately providing a better understanding of the lipid metabolic changes associated with pre-MetS and MetS. Plasma lipid profiles from 70 individuals were categorized into three groups: 28 with MetS, 28 with pre-MetS, and 14 healthy controls. Plasma samples were collected following a twelve-hour fast. This nontargeted approach identified 1,361 lipid metabolites, which underwent further processing and statistical analysis to determine differentially expressed lipids across the groups. A cross-sectional design was implemented, analyzing plasma samples from 70 participants: 28 MetS patients, 28 pre-MetS patients, and 14 healthy controls. Nontargeted lipidomic analysis was conducted using an UHPLC-MS (Courlet et al., 2020) coupled with a Qexactive Plus mass spectrometer (Huang et al., 2024). Lipid identification and quantification were carried out using LipidSearch software. Differentially expressed lipids were identified through OPLS-DA (Boccard & Rutledge, 2013), highlighting key lipid features. ML techniques, including Support Vector Machine Recursive Feature Elimination (SVM-RFE) (Lin et al., 2012), RF (Rigatti, 2017), and LASSO regression (Fontanarosa & Dai, 2011), were employed to identify significant lipid biomarkers. Model performance was evaluated using a ten-fold cross-validation approach and validated using metrics like AUC of the ROC curve (Çorbacıoğlu & Aksel, 2023).

The lipidomic analysis identified 1,361 lipid metabolites, of which 77 were significantly upregulated in pre-MetS patients compared to control samples, and 143 were significantly upregulated in MetS patients. ML feature selection further refined this list to identify key lipid biomarkers, such as *PS*(38:3) and *DG*(16:0/18:1) for pre-MetS, and *TG* (16:0/14:1/22:6), *TG* (16:0/18:2/20:4), and *TG* (14:0/18:2/18:3) for MetS (Huang et al., 2024). Among the models tested, LDA (Hu et al., 2020) showed the best performance for distinguishing pre-MetS from healthy controls, achieving an AUC of 0.89, while RF excelled in differentiating pre-MetS from MetS, with an AUC value of 0.88 (Huang et al., 2024). The identified lipid biomarkers provided significant discrimination between the groups, offering potential diagnostic markers for early detection of metabolic abnormalities (Huang et al., 2024). The study demonstrates the potential of integrating lipidomic with ML to differentiate between stages of metabolic syndrome, providing a non-invasive approach for early detection and risk stratification of pre-MetS and MetS. The identified lipid biomarkers offer insights into the metabolic disruptions occurring in these conditions, supporting the development of personalized treatment and prevention strategies. This approach could improve clinical decision-making and enable targeted interventions to mitigate the progression of MetS (Huang et al., 2024). However, the small sample size used in this study may limit the generalizability of its findings and poses a risk for overfitting in the ML models. Additionally, the lipidomic analysis was conducted using a single-center cohort from a specific geographic region, which may reduce the applicability of the results to broader populations. Further studies with larger, more diverse cohorts and external validation using multi-center cohorts are necessary to confirm the identified biomarkers’ diagnostic utility and the robustness of the predictive models.

### *CH25H* identified as asthma biomarker via ML (Ding et al., 2023)

The researchers of this study aimed to identify key lipid metabolism-related biomarkers associated with asthma (Papi et al., 2018) by integrating ML with an untargeted metabolomic approach. Specifically, the authors focused on identifying metabolites and genes that could serve as non-invasive biomarkers, with an emphasis on the lipid metabolism gene *Cholesterol 25-Hydroxylase (CH25H)*, which may play a role in asthma pathogenesis (Ding et al., 2023). The dataset comprised murine and human data drawn from public GEO datasets: 46 ovalbumin-induced murine asthma samples and 45 controls, alongside 62 human asthma patient samples and 43 healthy controls. Plasma samples were used to detect and quantify lipid metabolites, and gene expression data was sourced from relevant GEO datasets (GSE41665, GSE41667, GSE3184, GSE67472), allowing cross-validation across species and datasets. This study applied an untargeted metabolomics approach to identify potential lipid biomarkers for asthma, combining LC–MS (Seger & Salzmann, 2020) analysis with advanced ML algorithms (Ding et al., 2023). Plasma samples from both human and murine asthma datasets were processed using LC–MS, which allowed for the comprehensive profiling of lipid metabolites. The metabolite features were processed and normalized with PCA (Groth et al., 2013) and OPLS-DA (Boccard & Rutledge, 2013), which were used to assess sample clustering and significant lipid variations between asthma and control groups (Ding et al., 2023). LASSO (Fontanarosa & Dai, 2011) was employed to refine feature selection by filtering out less important variables, aiding in the identification of lipid biomarkers with strong predictive power (Ding et al., 2023). SVM-RFE (Lin et al., 2012) was applied to rank features based on importance, systematically removing those with lower contributions to enhance model efficiency (Ding et al., 2023). RF (Rigatti, 2017) and XGBoost (Moore & Bell, 2022) algorithms were used for classification, leveraging feature importance rankings to identify critical lipid metabolites and assess the model’s ability to differentiate between asthma and control samples (Ding et al., 2023). Additionally, the study conducted Weighted Gene Co-expression Network Analysis (WGCNA) (Zhang & Horvath, 2005) to explore the relationship between lipid metabolism and gene expression in asthma, specifically targeting genes linked to lipid metabolism such as *CH25H* (Ding et al., 2023). For pathway and functional enrichment analysis, R packages like WGCNA, limma, and clusterProfiler were used, with Cytoscape software utilized to visualize network associations among metabolites and gene expression data. This approach allowed for the integration of lipidomics and transcriptomics, pinpointing *CH25H* and other relevant genes.

Across all models, *CH25H* emerged as a central gene in lipid metabolism associated with asthma. Notable lipid biomarkers included *LPC 16:0*, *LPC 18:1*, and *LPA 18:1* (Ding et al., 2023). Among the models, RF achieved the highest predictive performance, with an AUC of 0.949, F1 score of 0.909, and accuracy of 0.938, outperforming other models such as SVM and XGBoost (Ding et al., 2023). These results support the robustness of RF in identifying meaningful lipid features associated with asthma. Importantly, ROC analysis demonstrated that *CH25H* alone achieved an AUC of 0.957 in the discovery dataset, indicating strong diagnostic accuracy and underscoring its potential as a standalone biomarker (Ding et al., 2023). *SGPP2*, another candidate gene, had a similar AUC of 0.946, but *CH25H* was prioritized because of its reproducibility across validation datasets. These metrics provide quantitative evidence that *CH25H* outperformed other candidates. Pathway analysis revealed lipid pathways, with key pathways involving phospholipid and lysophospholipid metabolism, as shown to be significantly altered in asthma patients.

This study demonstrated the value of integrating metabolomics with ML to uncover potential biomarkers and mechanisms in asthma. By identifying *CH25H* and specific lipid metabolites as relevant to asthma pathogenesis, the findings may inform future diagnostic and therapeutic strategies, providing a foundation for personalized treatment approaches targeting lipid metabolic pathways in asthma (Ding et al., 2023). The notably small sample size and reliance on public datasets introduce potential sources of variability in the findings. Specifically, the datasets were sourced from different platforms and included both murine and human samples, which may have biological variability and species-specific differences that complicate direct comparisons and translational relevance (Ding et al., 2023). For example, oxysterol regulation by *CH25H-*derived *25-hydroxycholesterol* has shown to modulate NF-kB-mediated inflammatory signaling differently in murine versus human lung tissue (Russell, 2000). Moreover, murine asthma models often feature predominantly eosinophilic inflammation, while human asthma encompasses more heterogenous immune endotypes, including metabolic and immunologic differences. These species-specific variations highlight the importance of validating *CH25H* in larger, well-characterized human data cohorts before it can be considered a reliable clinical biomarker. Although normalization techniques such as batch effect removal were applied, residual inconsistencies between datasets may persist.

## Discussion

**Fig. 2:**
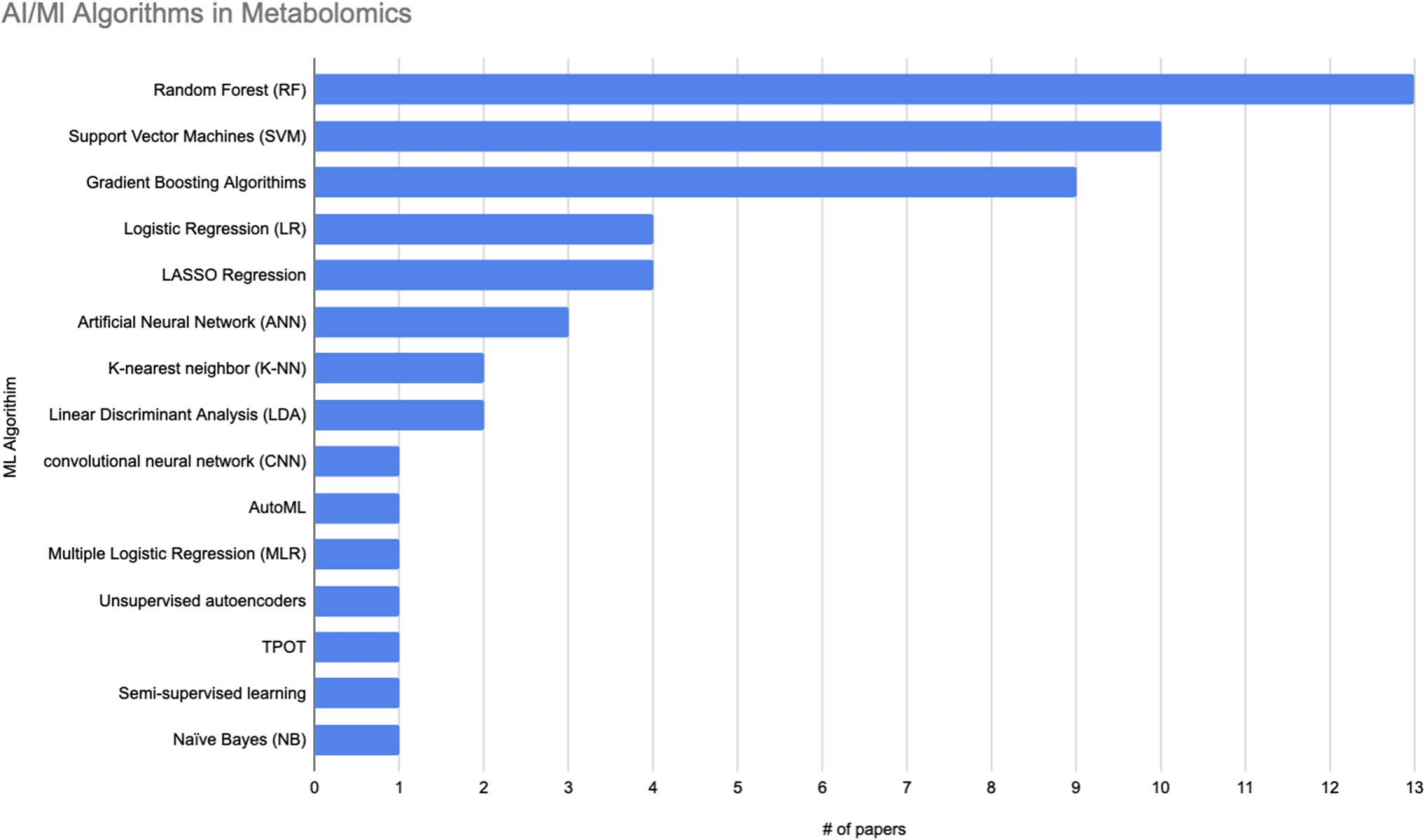
Most common AI/ML methods. AI/ML algorithms applied for predictive analysis, ranked in descending order of usage in our reviewed studies

The culmination of our state-of-the-art literature review resulted in our ability to assess the varying degrees of usage of various ML algorithms on metabolomic data in our reviewed studies (Fig. 2). These ML algorithms were ranked based on the number of studies utilizing each method. This ranking reveals distinct trends in adopting various ML approaches, influenced by their suitability for metabolomic data and study objectives. The comparison of AI/ML algorithms each specific study used is denoted in Table 1. The most frequently applied algorithms are RF, SVM, and Gradient Boosting Algorithms, cited in 12, 11, and 10 papers, respectively. These algorithms are useful for high-dimensional and complex datasets typically found in metabolomics. Their ability to handle non-linear relationships and provide feature-importance rankings makes them highly versatile for both classification and feature selection tasks (Bifarin & Fernández, 2024; Cielecka-Piontek et al., 2013; Lin et al., 2012; Moore & Bell, 2022; Natekin & Knoll, 2013; Shao et al., 2016). The dominance of tree-based methods like RF and Gradient Boosting is likely due to their accuracy across varied datasets. At the same time, SVM’s ability to find optimal decision boundaries in non-linear spaces contributes to its wide adoption (Lin et al., 2012; Noble, 2006).

Algorithms such as LR, LASSO Regression, and ANNs fall into a middle range of frequency, cited in 6, 5, and 4 papers, respectively. LR (Sperandei, 2014) and LASSO (Fontanarosa & Dai, 2011) regressions are favored for their simplicity and interoperability, which is crucial in studies aiming to identify potential biomarkers or understand specific metabolic processes. These regression-based methods are commonly used in early stages of data analysis to reduce dimensionality and focus on key variables. ANNs are not as commonly used in metabolomics, likely due to their computational demands and need for larger datasets (Huang et al., 2023). The least frequently applied methods included k-NN (Ehsani & Drabløs, 2020), LDA (Hu et al., 2020), CNNs (Alzubaidi et al., 2021), AutoML (Bifarin & Fernández, 2024), TPOT (Le et al., 2020), and Unsupervised Autoencoders (Baur et al., 2021), with only 1 to 3 studies citing their use. K-NN and LDA may struggle with metabolomics data's high-dimensional, noisy nature. CNNs are more suited to imaging and spatial data, which are less common in metabolomics studies (Alzubaidi et al., 2021). Emerging approaches like AutoML and TPOT show promise in automating and optimizing ML pipelines but remain underexplored in this field, likely due to the specialized expertise required for their application and the computational limits they often encounter with large datasets (Cerulli et al., 2021). The small dataset sizes and high interpretability demand of metabolomics are likely the main culprits for the rare usage of autoencoders, which are geared towards dimensionality reduction and anomaly detection in larger, more structured datasets (Baur et al., 2021).

The selection of algorithms for use on metabolomic datasets is closely tied to the features of these datasets, which are often characterized by high dimensionality, non-linear relationships, and small sample sizes. Algorithms like RF and Gradient Boosting are particularly adept at handling these challenges, making them reliable choices for many studies. Simpler algorithms, such as logistic regression, remain widely used because of their transparency and ease of implementation. In contrast, the lower adoption of methods such as Naive Bayes (Kaushik et al., 2022) and unsupervised autoencoders (Baur et al., 2021) may reflect their limitations in handling the complexity of non-linear metabolomics data or their focus on more niche applications. The application of AI/ML techniques on metabolomic data, although shown to be promising, is still underexplored due to the complexity and scarcity of metabolomic data. In the discovery stages of experimenting with different ML algorithms, researchers have mostly sought to find metabolic patterns in varying diseases, which could lead to uncovering potentially novel biomarkers (An et al., 2024; Anh et al., 2024; Chen et al., 2024; Ding et al., 2023; Drouard et al., 2024; Elgedawy et al., 2024; Huang et al., 2024; Künzel et al., 2024; Kuwabara et al., 2022; Lu et al., 2023; Moskaleva et al., 2022; Orlenko et al., 2020; Shah et al., 2023; Xu et al., 2024; Yagin et al., 2024; Zhang et al., 2024). This review led us to find that tree-based methods are most often employed by researchers looking for initial metabolic signatures of diseases, especially chronic diseases, likely due to the high accuracy and performance of these ML methods in complex datasets. These methods are often used in combination with logistic and LASSO regressions to reduce dimensionality and pinpoint key metabolic differences in patients with the disease from healthy patients (An et al., 2024; Anh et al., 2024; Cabello-Pinedo et al., 2024; Chen et al., 2024; Huang et al., 2024; Irajizad et al., 2022; Künzel et al., 2024; Kuwabara et al., 2022; Leiherer et al., 2024; Moskaleva et al., 2022; Zhang et al., 2024).

**Fig. 3:**
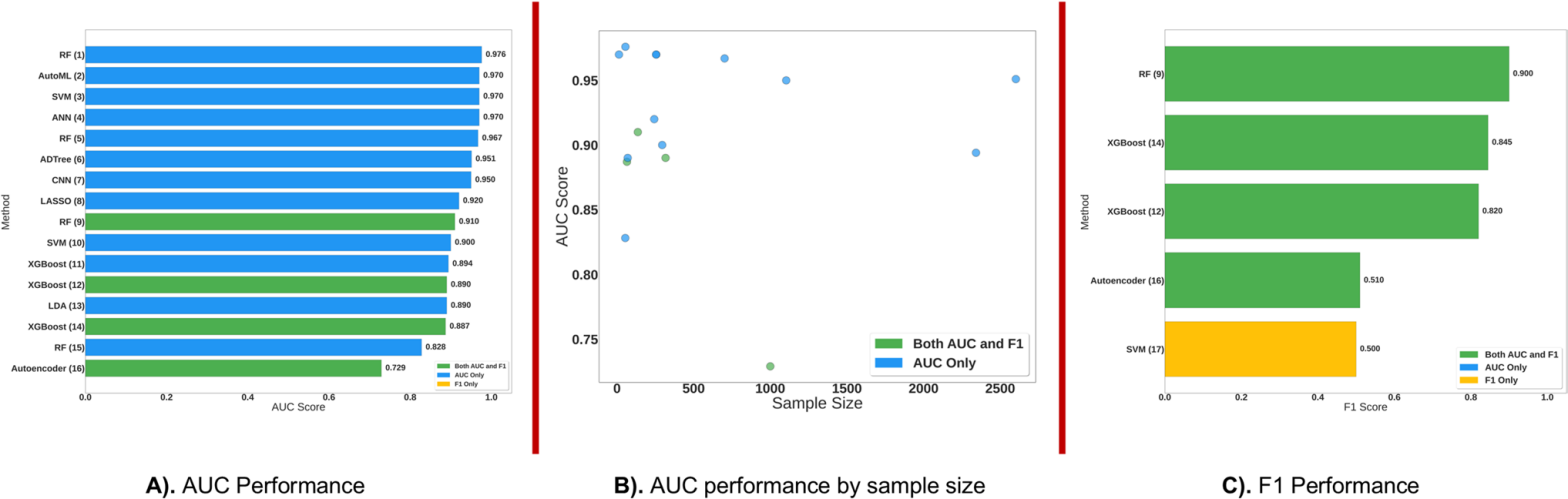
AUC and F1 performance metrics across included studies, alongside dataset population size for AUC scores. This figure presents ML metric performance across all studies that included these metrics in their methodology, compared to the dataset sizes for studies that used AUC scores. **A** AUC Performance across varying ML algorithms found in different referenced studies, where the y-axis is the algorithm used, and the x-axis is the AUC score, with a score of 1.0 representing a perfect ability to distinguish between healthy and diseased classes**.** The blue bars represent studies that only used an AUC score, the green bars represent studies that used both AUC and F1 scores, and the yellow bar (not included) are studies that only utilized an F1 score. **B** AUC performance compared to sample sizes used in reviewed studies, where the y-axis is the AUC score, and x-axis is the sample size used, ranging from n = 13 to n = 2602. **C** F1 performance metrics for the 5 studies that utilized this metric, where the y-axis is each study and the ML algorithm they used, and the x-axis is the F1 score, with a score of 1.0 representing perfect precision and recall between positive and actual positives. The green bars represent studies that used both F1 and AUC scores, the yellow bar is the study that only used an F1 score, and the blue bars (not included) represent studies that used only AUC scores

We noted the value of comparing algorithms in the reviewed studies based on their performance metrics, specifically the ML metrics AUC and F1 scores (Fig. 3). There were 16 studies that utilized AUC scores, where several model types, including RF, XGBoost, and SVM appear multiple times with different performance outcomes (Fig. 3A). The blue bars represent models from studies that only reported an AUC score; the green bars represent models from studies that reported both AUC and F1 scores. Among the 16 models, 12 were blue and 4 were green. The best performer was a RF model, with an AUC score of 0.976, followed closely by an AutoML model, an SVM, and an ANN, all tied with an AUC of 0.970. The middle of the ranking consisted of another RF (0.967), ADTree (0.951), CNN (0.950), LASSO (0.920), and additional instances of RF, SVM, XGBoost, and LDA with scores between 0.890 and 0.910 (Fig. 3A). At the lower end of the performance spectrum, the model with the lowest AUC score is the Autoencoder (0.729), with other models in the bottom portion of the rankings including an instance of RF (0.828) and an instance of XGBoost (0.887). We also looked at the AUC performance compared to dataset sizes, with the lowest dataset being n = 13 and the largest being n = 2602 (Fig. 3B). Many of the top performing models had small sample sizes (n < 500). This increases the risk of overfitting and reduces model generalizability. However, there were also some models with large sample sizes (n > 2000) which reported high AUC scores, such as an ADtree model which had a sample size of n = 2602 and achieved 0.951 AUC. An XGBoost model with sample size n = 2342 achieved a 0.894 AUC. The lowest AUC performer was an Autoencoder which had a moderate sample size of n = 1000. The top performing AUC model was a RF model with a sample size of n = 55. These high scores exemplify the value of using large datasets, with high AUC performance metrics but also greater generalizability and real-world applications. It also warns the use of smaller datasets; even though there was high performance on such datasets, it may not perform well on new data or more general and diverse datasets due to overfitting. We hypothesize that small sample sizes (e.g., n approx. 50) can yield optimistically high AUCs due to variance and potential overfitting, even when cross-validation is used. Lastly, we observed the performance of F1 values across all studies that used this metric (Fig. 3C). We noted 5 ML models in order of decreasing F1 score: RF, XGBoost, Autoencoder, and SVM. The green bars represent models from studies that reported both AUC score and F1 score. The yellow bar represents models from studies that only reported an F1 score. Among the 5 models, 4 were green and 1 was yellow. RF and XGBoost were the top performers while Autoencoders and SVM produced poor results (Fig. 3C). The top performers for both F1 and AUC scores were RF models, and at the same time, RF was also one of the most frequently used algorithms, proving both its implementation abilities and performance on metabolic datasets (Fig. 2 and Fig. 3).

However, there are three limitations involved with AI applications on metabolomic data: 1) limited data arising from small cohort sizes in trials, 2) high computational costs due to complex algorithms, and 3) hefty storage needs for those studies that have large and compounded datasets or for source datasets in transfer learning. For most of these studies, larger trials with metabolomic data from more patients need to be conducted to validate current findings (Anh et al., 2024; Cabello-Pinedo et al., 2024; Drouard et al., 2024; Elgedawy et al., 2024; Huang et al., 2024; Künzel et al., 2024; Moskaleva et al., 2022; Orlenko et al., 2020; Shah et al., 2023; Shen et al., 2024; Zhang et al., 2024). If there isn’t enough data, outliers and missing value imputation may be introduced due to the heterogenous and noisy nature of metabolomic data, and using larger datasets helps combat this (Ahmed et al., 2024). Approaches such as MetSizeR have been developed to estimate metabolomic sample size requirements on a case-to-case basis, simulating pilot data and considering the type of analysis the researcher is intending to perform on the metabolomic data; applications such as MetSizeR are increasingly imperative given the lack of standard protocols for sample size determination (Nyamundanda et al., 2013). In general, guidelines suggest that model training should be based on a minimum sample size that is sufficient to approximate the overall population risk; therefore, the mean predicted risk should align with the mean observed risk in target populations, and datasets that fail to meet these criteria should not be used for models intended to be used for individual risk prediction (Riley et al., 2025). Overfitting also becomes a challenge with models trained on small, where a model performs well on the training data but fails to analyze new data (Ahmed et al., 2024). Indeed, smaller datasets produce great instability in model predictors and contribute to lower predictive performance compared to models trained on larger sample sizes (Sarker, 2021). The weaker predictive performance can be due, in part, to improper data splitting, blurring the lines between training and validation, causing models to potentially work with data during the testing phase that should be left for the testing phase, which can lead to weaker real-world predictions (Sivakumar et al., 2024). Additionally, larger datasets can work with more complex AI/ML model applications leading to stronger predictive power, as seen in datasets like MassSpecGym, which incorporates the largest publicly available collection of high-quality labeled MS/MS spectra (samples in the tens of thousands) (Bushuiev et al., 2025a).

Datasets such as these are more conducive to deep learning methods like artificial neural networks, leading to stronger predictive power and clinical applications (Sarker, 2021; Vadapalli et al., 2022). Smaller datasets also pose a risk for application and reproducibility, but already, researchers have found ways to improve reproducibility by using virtual environments like Conda, addressing the limitations of past computational metabolomics tools such as monolithic software which hinders high throughput analysis due to the inability to scale individual layers, or using proprietary closed-source solutions, which create a barrier for reproducibility (Perez-Riverol & Moreno, 2020). Using BioConda packages automates deployment of tools for analysis within the software, providing a stable software environment, cross-platform uniformity, and support of scalable systems (Perez-Riverol & Moreno, 2020). Despite these recently developing frameworks to improve reproducibility, most of the current AI metabolomic models deduced from our review would still benefit from being trained on larger datasets to ensure capturing metabolomic signatures from a diverse population, thereby reducing bias, improving accuracy, reducing the likelihood of overfitting, and eliminating potential outliers before applying findings to clinical use. However, before urging studies to utilize large datasets, it is important to recognize that large datasets carry challenges of their own: researchers face computational and storage costs because algorithms need high computational power and large storage capacity to save logs, results, and analysis (Ahmed et al., 2024).

A potential mitigation to addressing the limitations of inherently small datasets while also addressing the challenge of storage needed to work with large datasets is transfer learning using compact storage techniques. Transfer learning is a technique already widely used in ML research. It can use the feature and pattern recognition of ML models trained on larger, more complex sets, and fine-tune the trained learnings to smaller, more specific datasets as found in many of the reviewed metabolic studies (Kowald et al., 2022). This can reduce the risk of overfitting or outputting spurious correlations deduced from memorization instead of authentic pattern learning. Bushuiev et al. have showcased a unique technique to efficiently utilize transfer learning while accounting for storage challenges that come with large source data. In their study, they mine spectra data, filtering and clustering for redundancy, and then further channel that through a HDF5-binary format, allowing for compact storage and easier access for deep learning (Bushuiev et al., 2025b). They further use these large source datasets to perform self-supervised pre-training of their model to predict spectral peaks and chromatographic retention orders, leading to holistic insights and deep representations of molecular structures (Bushuiev et al., 2025b). These pre-trained neural network models are then fine-tuned to perform a variety of tasks on smaller target datasets, eliminating risks of overfitting or inauthentic ML outputs. Even though working with metabolomic data requires high expertise and costs, researchers have already found novel metabolic patterns, showing the potential that metabolomic data holds in contributing to precision medicine and uncovering key metabolic pathways related to a plethora of diverse diseases. For example, tryptophan metabolomic biomarkers have been used to predict hepatocellular carcinoma in a study performed by Xue et al. (Xue et al., 2022). This study elucidated two distinct metabolic phenotypes, or patient clusters, based on expression of 40 significant prognostic Trp-metabolism genes, where cluster 1 encapsulated incorrect prognosis patients, and cluster 2 was better prognosis. The researchers also built a 10-gene risk model that can be used to stratify patients into risk clusters based on biomarker expression levels, propelling clinical decision making and appropriate treatment options based on distinct immunotherapy response predictions across the 2 groups (Xue et al., 2022). Furthermore, the translational pathway of using the protein biomarker Galectin-3 for clinical use in potentially treating metabolic dysfunction-associated Fatty Liver disease, which can give rise to more severe diseases such as liver fibrosis (Sotoudeheian, 2024). These studies highlight real-world translational pathway execution, although they do not heavily rely on the use of ML models to support metabolomic data analysis.

AI models that combine metabolomic data with other omics such as genomics and proteomics data have the potential to pinpoint disease causes on an individual basis, create novel drugs based on newly discovered pathways, and develop a more complete understanding of disease manifestations and etiology. Multiple researchers such as Drouard et al., and Liu et al., have already started corroborating multi-omics in their studies, and have found correlations between genomic, proteomic and metabolomic data (Drouard et al., 2024; Liu et al., 2021a). Researchers found that models built from multi-omics data performed better in most scenarios, but for certain diseases where the phenotype predominates in one type of -omics data, single omics data was found to be on par with multi-omics methodology (Drouard et al., 2024). Researchers seeking to implement a multi-omics approach face another set of challenges since they are working with a larger number and different sets of high throughput data. More dimensionality is introduced to the data, thereby requiring more complex algorithms to simplify the data for the model, which further increases computational and storage costs (Ahmed et al., 2024). In our attempts to discover multi-modal AI/ML applications that are reproducible, transparent, use next-generation AI/ML techniques, highly interactive and collaborative, and communicative of uncertainty and errors, we have seen great progress towards achieving these pillars, but notice valuable room for improvement. In the future, AI/ML applications intended for precision medicine and translational sciences can improve upon integrating more multi-omics layers beyond just metabolomic data, incorporating larger datasets or maximizing the use of transfer learning, and instituting more transferrable/reproducible frameworks. Ultimately, researchers continue to build AI models with omics data because of the possibilities to reveal a novel and more nuanced understanding of disease etiology, diagnosis potential, underlying pathways, and patient response to treatment, ultimately leveraging the medical field.

## Data Availability

No datasets were generated or analysed during the current study.

## Abbreviations

AdaBoost: Adaptive boosting
AD: Adenoma
ADP: Adenosine diphosphate
AMP: Adenosine monophosphate
DKD-A: Advanced-stage diabetic kidney disease
ADTree: Alternating decision tree
ANOVA: Analysis of variance
anti-VEGF: Anti-vascular endothelial growth factor
AUC: Area under the curve
AI: Artificial intelligence
ANN: Artificial neural network
AF: Atrial fibrillation
BNB: Bernoulli naïve bayes
BMI: Body mass index
C4: Butyrylcarnitine
CCA: Canonical correlation analysis
CE-MS: Capillary electrophoresis-mass spectrometry
CDCA: Chenodeoxycholic acid
CHOL: Cholesterol
CH25H: Cholesterol 25-hydroxylate gene
CNV: Choroidal neovascularization
CAC: Chronically active CNV
CAD: Coronary artery disease
CHD: Coronary heart disease
DCA: Deoxycholic acid
DKD: Diabetic kidney disease
DR: Diabetic retinopathy
DBP: Diastolic blood pressure
DEM: Differentially expressed metabolites
DEP: Differentially expressed proteins
DMA: Dimethylarginine
DKD-E: Early-Stage diabetic kidney disease
ECC: Effectively controlled CNV
ESC: Esophageal squamous cell carcinoma
eGFR: Estimated glomerular filtration rate
XAI: Explainable artificial intelligence
EBM: Explainable boosting machine
XGBoost: Extreme gradient boosting
FDR: False discovery rate
FASP: Filter-aided sample preparation
FIA-MS: Flow injection analysis
FA: Fluorescein angiography
FC: Fold change
FAF: Fundus autofluorescence
F1 Score: F1 score
GC–MS: Gas chromatography-tandem mass spectrometry
GC: Gastric cancer
GSEA: Gene set enrichment analysis
GDM: Gestational diabetes mellitus
C5DC: Glutarylcarnitine
Hb12Ac: Glycated hemoglobin
HR: Hazard ratio
HC: Healthy controls
HDL-C: High-density lipoprotein cholesterol
HMDC: Human metabolite database
HTA: Hypertension
IRC: Intraretinal cysts
IVT: Intravitreal therapy
IVF: In-vitro fertilization
KEGG: Kyoto encyclopedia of genes and genomes
LASSO: Least absolute shrinkage and selection operator
LVDD: Left ventricular diastolic dysfunction
LightGBM: Light gradient boosting machine
LDA: Linear discriminant analysis
LOESS: Locally weighted scatterplot smoothing
LR: Logistic regression
LDL-C: Low-density lipoprotein cholesterol
LPA: Lysophosphatidic acid
LPC: Lysophosphatidylcholine
LPE: Lysophosphatidylethanolamine
ML: Machine learning
MetS: Metabolic syndrome
MPI: Metabolite pregnancy index
mRMR: Minimum redundancy maximum relevance
MLR: Multiple linear regression
MI: Myocardial infarction
NPC: Nasopharyngeal carcinoma
NMDR: National metabolomics data repository
NGBoost: Natural gradient boosting
NPV: Negative predictive value
NACT: Neoadjuvant chemotherapy
nAMD: Neovascular age-related macular degeneration
NDR: Non-diabetic retinopathy
NGC: Non-gastric cancer
NPDR: Non-proliferative diabetic retinopathy
NTM: Nontuberculous mycobacteria
NMR: Nuclear magnetic resonance
OCT: Optical coherence tomography
OT-FTMS: Orbitrap fusion tribrid mass spectrometer
OPLS-DA: Orthogonal partial least squares discriminant analysis
OC: Ovarian cancer
PLS-DA: Partial least squares discriminant analysis
PA: Phosphatidic acid
PC: Phosphatidylcholine
PC.aa.C42.2: Phosphatidylcholine diacyl C42:2
PE: Phosphatidylethanolamine
PI: Pooled intensity
PPV: Positive predictive value
Pre-MetS: Pre-metabolic syndrome
PCA: Principal component analysis
PDR: Proliferative diabetic retinopathy
RF: Random forest
E/A Ratio: Ratio of early to late ventricular filling
ROC: Receiver operating characteristic
RFE: Recursive feature elimination
RCC: Renal cell carcinoma
SN: Screening-negative
SP: Screening-positive
SHAP: Shapley additive explanations
SRF: Subretinal fluid
SHRM: Subretinal hyperreflective material
SVM: Support vector machine
SVM-RFE: Support vector machine recursive feature elimination
SVR: Support vector regression
SMOTE: Synthetic minority oversampling technique
SBP: Systolic blood pressure
TMT: Tandem mass tag
TPOT: Tree-based pipeline optimization tool
TG: Triglycerides
TNBC: Triple-negative breast cancer
TSQ: Triple quadrupole mass spectrometer
TrP: Tryptophan
TB: Tuberculosis
T2D: Type 2 diabetes
Tyr: Tyrosine
UHPLC-MS: Ultra-high performance liquid chromatography-mass spectrometry
UPLC-MS: Ultra-performance liquid chromatography-mass spectrometry
WGCNA: Weighted gene co-expression network analysis
